# Supplementary material for: An in vivo strategy to counteract post-administration anticoagulant activity of azido-Warfarin
Source: Nat Commun. 2017 May 19;8:15242. doi: 10.1038/ncomms15242 (PMC5454458; doi:10.1038/ncomms15242)
Supplement: Supplementary Information — Supplementary Figures, Supplementary Methods and Supplementary References [file ncomms15242-s1.pdf]

Supplementary Methods

Material and methods

Experimental procedures. Unless otherwise indicated, reactions were carried out under an argon atmosphere in flame-dried glassware with magnetic stirring. Air and/or moisture-sensitive liquids were transferred *via* syringe. When required, solutions were degassed by argon bubbling through a needle. Organic solutions were concentrated by rotary evaporation at 25-80 °C and 15-30 torr. Analytical thin layer chromatography (TLC) was performed using plates cut from aluminium sheets (ALUGRAM Xtra SIL G/UV_{254} from Macherey-Nagel). Visualization was achieved under a 254 or 365 nm UV light and by immersion in an appropriate revelation solution.

Materials. All reagents used in the experiments were purchased from Aldrich, Alfa Aesar, Acros or TCI and were used without any further purification. Anhydrous solvents used in experiments were obtained from Sigma-Aldrich or Alfa Aesar. Silica gel for column chromatography was purchased from Merck (Geduran® Si 60, 40-63 µm). Column flash chromatography was carried out using silica gel G-25 (40-63 µm) from Macherey-Nagel.

Instrumentation and methods associated

NMR spectroscopy, ^{1}H and ^{13}C NMR spectra were recorded at 23 °C on a Bruker 400 spectrometer. Recorded shifts are reported in parts per million (δ) and calibrated using residual non-deuterated solvent. Data are represented as follows: chemical shift, multiplicity (s = singlet, d = doublet, t = triplet, q = quartet, quint = quintet, m = multiplet, br = broad), coupling constant (*J*, Hz) and integration.

Analytical HPLC experiments were carried out on a Shimadzu system (pump: LC 20-AD, detector: SPD 20-A, autosampler: SIL 20-A) using a SunFire™ C18 5 µM 4.6 × 150 mm column (Waters). HPLC parameters were as follows: flow rate 1 mL/min, gradient from 5 to 95% of mobile phase B from 0 to 20 min, followed by 5 min at 95% of mobile phase and post time of 5 min. Mobile phase A was 0.05% TFA in water (mQ) (v/v), and mobile phase B was acetonitrile (HPLC grade). The detection was done at 306 nm for kinetic studies of the SPAAC reaction.

High-resolution mass spectra (HRMS) were obtained using an Agilent Q-TOF (time of flight) 6520.

Low-resolution mass spectra were obtained using an Agilent MSD 1200 SL (ESI/APCI) with a, Agilent HPLC1200 SL and a Waters Acquity QDa (ESI) with a Waters Alliance 2695 HPLC.

LC-MS/MS metabolomic analyses were performed using a Triple Quadrupole Liquid Chromatograph Mass Spectrometer (LCMS 8030, Shimadzu) in multiple reaction-monitoring mode (MRM). Data acquisition and processing were performed using LabSolutions version 5 software. Measurements were carried out at 40°C. The mobile phase flow rate was fixed at 0.5 mL/min and the following program was applied for the elution: 0 min, 5% B; 0-1.2 min, 5-95% B; 1.2-1.4 min, 95% B; 1.4-1.42 min, 95-5% B and 1.42-2.8 min, 5% B. Solvent A consisted of 0.05% formic acid in water and solvent B was HPLC grade acetonitrile. Injection volume was 1 µL. The mass spectrometer was interfaced with the liquid chromatograph using an electrospray ion source. The nitrogen nebulizing gas flow was set at 1.5 L/min and the drying gas flow at 15 mL/min. 4500 V were used for the interface voltage. The temperature of the block heater was maintained at 400 °C and the one of the desolvation line at 250 °C. The MRM transitions were 348.1 → 263.1 / 320.1, 475.4 → 45.1 / 89.1 and 807.4 → 454.2 / 250.3, respectively for WN_3 **2**, BCN-peg_6-OH **9** and clicked product **10**. The collision gas used was argon at 230 kPa. The dwell time was set to 100 msec and the pause time to 3 msec.

Prothrombin time analyses were performed using system STart4® instrument (Diagnostica Stago).

Chemical synthesis

Synthesis of WN_3 (2) derivative

The synthesis towards WN_3 **2** started with substitution of *para*-nitrobenzaldehyde **3** by sodium azide in DMF under microwave irradiation to afford *para*-azidobenzaldehyde **4**. Following the procedure described by Zumbassen *et al.*,[1] a Knoevenagel condensation was performed between *para*-nitrobenzaldehyde **4** and acetone catalyzed by morpholinium trifluoroacetate to give (*E*)-4-(4-azidophenyl)but-3-en-2-one **5** in 63% yield. Finally, this α,β-unsaturated ketone was submitted to a Michael reaction with 4-hydroxycoumarine catalyzed by *S*-proline.[2] 3-(1-(4-azidophenyl)-3-oxobutyl)-2*H*-chromen-2-one (WN_3 **2**) was thus prepared in three steps with a 25% overall yield.

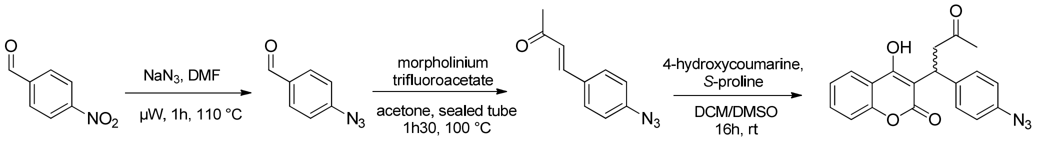

3 **4 (73%)** **5 (63%)** **2 (55%)**, WN_3

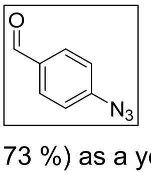

4-azidobenzaldehyde[3] (4). 4-nitrobenzaldehyde **3** (1 eq., 500 mg, 3.31 mmol) and NaN_3 (2 eq., 430 mg, 6.62 mmol) were dissolved in DMF (20 mL) in a microwave tube and stirred at 110°C under µW for 1h. The mixture was diluted with water (150 mL), and extracted 3 times with DCM (3×100 mL). The organic layer was washed with brine (100 mL), dried over $MgSO_4$ and evaporated. The crude material was purified by silica gel flash chromatography (Cyclohexane to EtOAc in 15 min) to give 4-azidobenzaldehyde **4** (355 mg, 2.42 mmol, 73 %) as a yellowish oil. **^{1}H NMR (400 MHz, $CDCl_3$-*d*)**: δ (ppm) 9.95 (s, 1H), 7.89 (d, *J* = 8.6 Hz, 2H), 7.17 (d, *J* = 8.6 Hz, 2H). **^{13}C NMR (100 MHz, $CDCl_3$-*d*)**: δ (ppm) 190.6, 146.2, 133.2, 131.5, 119.5.

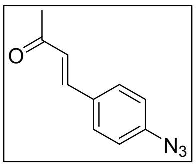

(*E*)-4-(4-azidophenyl)but-3-en-2-one (5). To a solution of 4-azidobenzaldehyde **4** (1 eq., 500 mg, 3.40 mmol) in acetone (3.4 mL) was added morpholinium trifluoroacetate (0.2 eq., 136 mg, 0.68 mmol). The reaction mixture was stirred in a sealed vial for 1h30 at 100°C. The acetone was evaporated and the crude material was purified by silica gel flash chromatography (Cyclohexane to EtOAc in 15 min) to give (*E*)-4-(4-azidophenyl)but-3-en-2-one **5** (400 mg, 2.14 mmol, 63 %) as a yellow powder. Morpholinium trifluoroacetate was synthesized according to the procedure described in the literature.[4] **^{1}H NMR (400 MHz, MeOD-*d$_4$*)**: δ (ppm) 7.70 (d, *J* = 8.5 Hz, 2H), 7.64 (d, *J* = 16.3 Hz, 1H), 7.13 (d, *J* = 8.5 Hz, 2H), 6.77 (d, *J* = 16.3 Hz, 1H), 2.39 (s, 3H). **^{13}C NMR (100 MHz, MeOD-*d$_4$*)**: δ (ppm) 201.8, 144.6, 143.9, 133.0, 131.4, 127.6, 120.8, 27.9. **MS (ESI, m/z)**: $[M+Na]^+$ = 210.

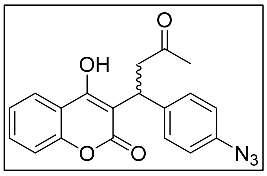

3-(1-(4-azidophenyl)-3-oxobutyl)-4-hydroxy-2*H*-chromen-2-one (WN_3, 2). (*E*)-4-(4-azidophenyl)but-3-en-2-one **5** (1 eq., 973 mg, 5.20 mmol) and 4-hydroxycoumarin (1.08 eq., 910 mg, 5.62 mmol) were dissolved in DCM (35 mL). A solution of *S*-proline (50 %, 299 mg, 2.6 mmol) in DMSO (15 mL) was added and the reaction mixture was stirred at room temperature overnight. Dichloromethane was removed under vacuum and the mixture was diluted with water (150 mL) then extracted with EtOAc (150 mL). The organic layer was washed with brine, dried over $MgSO_4$ and evaporated.

The crude material was purified by silica gel flash chromatography (Cyclohexane to EtOAc in 15 min) to give WN_3 **2** (999 mg, 2.86 mmol, 55 %) as a beige solid.

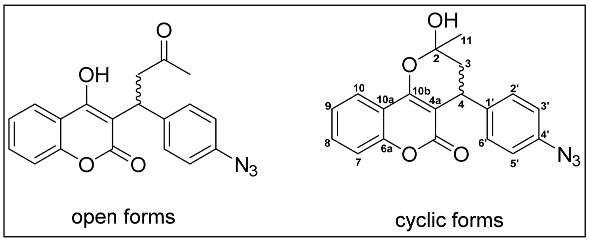

As the parent warfarin **1a**,[5] WN_3 **2** in solution exists in a dynamic equilibrium between the open form and two diastereomeric cyclic forms. In acetone only the two diastereoisomeric hemiketals were observed by ^1H and ^{13}C NMR. **^1H NMR (500MHz, Acetone-*d*$_6$)**: δ (ppm) 7.95 - 7.87 (m, 1H, H-10), 7.71 - 7.62 (m, 1H, H-8), 7.44 - 7.31 (m, 4H, H-7,9,18,22), 7.03 (d, *J*=8.5 Hz, 1.4H, H-3',5'), 6.98 (d, *J*=8.5 Hz, 0.6H, H-3',5'), 6.41 (br. s, 0.7H, OH), 6.17 (br. s, 0.3H, OH), 4.19 - 4.12 (m, 1H, H-4), 2.44 - 2.52 (m, 1.1H, H-3), 2.33 - 2.40 (m, 0.3H, H-3), 2.04 - 2.06 (m, 0.4H, H-3), 2.03 - 2.02 (m, 0.2H, H-3), 1.79 (s, 2.1H, H-11), 1.74 (s, 0.9H, H-11). **^{13}C NMR (126MHz, Acetone-*d*$_6$):** δ (ppm) 161.7 (C=O-5), 161.1 (C=O-5), 160.3 (C-O-10b), 159.7 (C-O-10b), 154.0 (C-6a), 153.9 (C-6a), 142.4 (C-1'), 142.2 (C-1'), 138.6 (C-4'), 138.3 (C-4'), 132.7 (CH-8), 132.6 (CH-8), 130.2 (CH-2',6'), 129.8 (CH-2',6'), 124.7 (CH-9), 124.6 (CH-9), 123.8 (CH-10), 119.9 (CH-3',5'), 119.4 (CH-3',5'), 117.2 (C-10a), 117.1 (CH-7), 117 (CH-7), 116.9 (C-10a), 104.9 (C=O-2), 103.2 (C=O-2), 101.8 (C-4a), 100.4 (C-4a), 43.7 (CH2-3), 41.9 (CH2-3), 36.6 (CH-4), 36.2 (CH-4), 28.0 (CH3-11), 27.1 (CH3-11). **HRMS (ESI, m/z):** calcd for $C_{19}H_{16}N_3O_4$ $[M+H]^+$ 350.11354; found 350.11358.

Synthesis of BCN-peg$_6$-OH (9) derivative

BCN was first activated as a *para*-nitrophenol carbonate according to the procedure described by Dommerholt, J. *et al.* [6] and then reacted with a prealably prepared amino oligoethylene derivative **8** synthesized in three steps starting from hexaethylene glycol **11**.

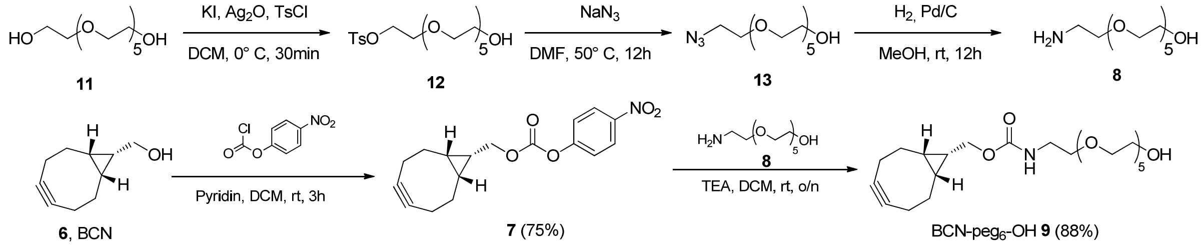

17-{[(4-methylphenyl)sulfonyl]oxy}-3,6,9,12,15-pentaoxaheptadecan-1-ol [7] **(12).** To a solution of

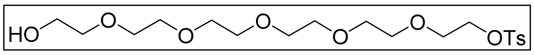

hexaethylene glycol **11** (1 eq., 7.88 g, 27.9 mmol) in DCM (315 mL) at 0°C were added KI (0.1 eq., 0.463 g, 0.309 mL, 2.79 mmol) and Ag_2O (1.2 eq., 7.76 g, 33.5 mmol). Tosyl chloride (1.05 eq., 5.59 g, 29.3 mmol) was then added by portion and the reaction mixture was stirred at 0°C for 30 minutes. The mixture was filtered through a pad of celite and concentrated. The crude material was purified by silica gel flash chromatography (DCM to DCM/MeOH 95/05) to give **12** (9.46 g, 21.7 mmol, 78 %) as a clear yellow oil. **^1H NMR (400 MHz, $CDCl_3$)**: δ 7.77 (d, *J* = 8.2 Hz, 2H), 7.32 (d, *J* = 8.0 Hz, 2H), 4.14 (t, *J* = 4.8 Hz, 2H), 3.72 – 3.53 (m, 22H), 2.42 (s, 3H), the OH signal is missing. **^{13}C NMR (100 MHz, $CDCl_3$)**: δ 144.8, 133.1, 129.8 (2C), 128.0 (2C), 72.6, 70.7-70.3, 69.3, 68.7, 61.7, 21.6.

17-azido-3,6,9,12,15-pentaoxaheptadecan-1-ol[8] (13). To a mixture of **12** (1 eq., 9.1 g, 20.8 mmol) in 25 mL of DMF was added NaN_3 (1.5 eq., 2.03 g, 31.3 mmol). The reaction was stirred at 50°C for 5h. After evaporation, 100 mL of water were added and the solution was extracted with DCM (3 × 150 mL). The organic layer was dried ($MgSO_4$) and concentrated in vacuo. The crude material was purified by silica gel flash chromatography (EtOAc to EtOAc/MeOH 9/1 in 25 min) to afford **13** (6.15 g, 20 mmol, 96 %) as a yellow oil. **^{1}H NMR (400 MHz, $CDCl_3$)**: δ 3.74 – 3.47 (m, 22H), 3.32 (d, *J* = 5.0 Hz, 2H), 2.77 (t, *J* = 6.1 Hz, 1H). **^{13}C NMR (100 MHz, $CDCl_3$)**: δ 72.5, 70.7 – 70.6, 70.4, 70.0, 61.8, 50.7.

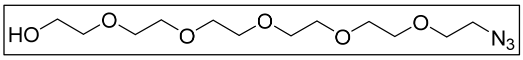

17-amino-3,6,9,12,15-pentaoxaheptadecan-1-ol[9] (8). To a solution of **13** (1 eq., 390 mg, 1.270 mmol) in MeOH (45 mL) was added Pd/C (2 %, 27 mg, 0.025 mmol) and the reaction mixture was stirred at room temperature under atmospheric pressure of hydrogen. After 12h, DCM was added and the mixture was filtered through a pad of celite. After concentration, the crude material was purified by silica gel flash chromatography (DCM to DCM/MeOH/NH_4OH 85/14/1) to afford **8** (292 mg, 1.04 mmol, 82 %) as a yellow oil. **^{1}H NMR (400 MHz, $CDCl_3$)**: δ 3.74 – 3.50 (m, 22H), 3.32 (d, *J* = 5.0 Hz, 2H), 2.77 (t, *J* = 6.1 Hz, 1H). The NH_2 signal is missing.**^{13}C NMR (100 MHz, $CDCl_3$)**: δ δ 73.1, 72.8, 70.6 – 70.5, 70.3, 70.2, 61.3, 41.5.

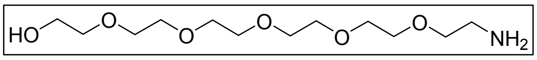

(1*R*,8*S*,9*S*)-bicyclo[6.1.0]non-4-yn-9-ylmethyl (4-nitrophenyl) carbonate (7). To a solution of BCN **6** (1 eq., 200 mg, 1.43 mmol) and pyridine (5 eq., 566 mg, 0.579 mL, 7.16 mmol) in DCM (7 mL) was added *para*-nitrophenol chloroformate (1.5 eq., 432 mg, 2.15 mmol). After stirring for 30 minutes at room temperature, 50 mL of an aqueous solution of NaH_2PO_4 were added and and the solution was extracted with Et_2O (3 x 50 mL). The combined organic layer was dried over $MgSO_4$ and concentrated. The crude material was purified by silica gel flash chromatography (cyclohexane to cyclohexane/EtOAc 9/1 in 20 min) to afford (1*R*,8*S*,9*S*)-bicyclo[6.1.0]non-4-yn-9-ylmethyl (4-nitrophenyl) carbonate **7** (400 mg, 1.27 mmol, 89 %) as a white solid. **^{1}H NMR (400 MHz, $CDCl_3$)**: δ 8.18 – 8.10 (m, 2H), 7.29 – 7.21 (m, 2H), 4.26 (d, *J* = 8.3 Hz, 2H), 2.25 – 2.07 (m, 6H), 1.52 – 1.32 (m, 3H), 0.99 – 0.84 (m, 2H). **^{13}C NMR (100 MHz, $CDCl_3$)**: δ 155.6, 152.6, 145.4, 125.3, 121.8, 98.7, 68.0, 29.1, 21.4, 20.5, 17.3.

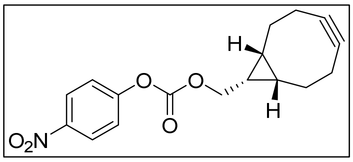

(1*R*,8*S*,9*s*)-bicyclo[6.1.0]non-4-yn-9-ylmethyl(17-hydroxy-3,6,9,12,15-pentaoxaheptadecyl) carbamate (BCN-peg$_6$-OH, 9). (1*R*,8*S*,9*s*)-bicyclo[6.1.0]non-4-yn-9-ylmethyl (4-nitrophenyl) carbonate **7** (1 eq., 200 mg, 0.634 mmol) was dissolved in DMF (2 mL). A solution of **14** (1.5 eq., 267 mg, 0.951 mmol) and TEA (3 eq., 192 mg, 0.264 mL, 1.9 mmol) in DMF (1 mL) was then added and the reaction was stirred at room temperature overnight. Completion of the reaction was checked by TLC (EtOAc/MeOH 95/5, Revelator: PMA or $KMnO_4$). After evaporation, 30 mL of water was added and the solution was extracted with EtOAc (3 x 50mL). The crude was purified by flash chromatography (Si 15g, DCM to DCM/MeOH 85/15) to afford BCN-peg$_6$-OH **9** (247 mg, 0.54 mmol, 85 %) as a yellowish oil. **^{1}H NMR (400 MHz, $CDCl_3$):** δ (ppm) 5.39 (s, 1H), 4.13 (d, *J* = 7.9 Hz, 2H), 3.72 – 3.59 (m, 20H), 3.54 (t, *J* = 5.0 Hz, 2H), 3.37 – 3.35 (m, 2H), 2.72 (bs, 1H), 2.31 – 2.18 (m, 6H), 1.58 – 1.56 (m, 2H), 1.37 – 1.32 (m, 1H), 0.95 – 0.90 (m, 2H). **^{13}C NMR (125 MHz, $CDCl_3$)**: δ (ppm) 156.9, 98.8, 72.6, 70.6 – 70.5, 70.3, 70.2, 70.2, 62.6, 61.7, 40.8, 29.1, 21.4, 20.1, 17.8. **HRMS (ESI, m/z):** calcd for $C_{23}H_{40}NO_8$ $[M+Na]^+$ 480.2568; found 480.2578.

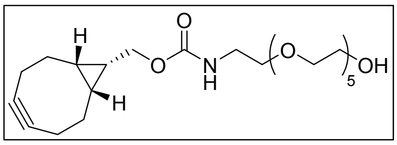

Synthesis of clicked product (10)

SPAAC reaction was finally performed using WN_3 **2** and BCN-peg$_6$-OH **9** in MeOH to afford the clicked product **10** in 98% yields (HPLC).

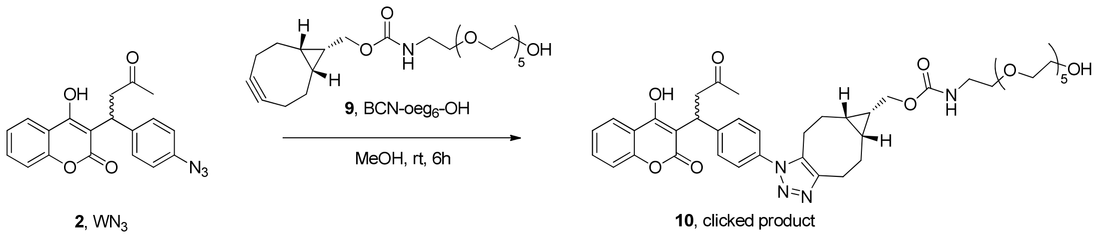

((5a*R*,6*S*,6a*S*)-1-(4-(1-(4-hydroxy-2-oxo-2*H*-chromen-3-yl)-3-oxobutyl)phenyl)-1,3a,4,5,5a,6,6a,7,8,8a-decahydrocyclopropa[5,6]cycloocta[1,2-*d*][1,2,3]triazol-6-yl) methyl (2-(2-ydroxyethoxy)ethyl) carbamate, 10 (Clicked product).

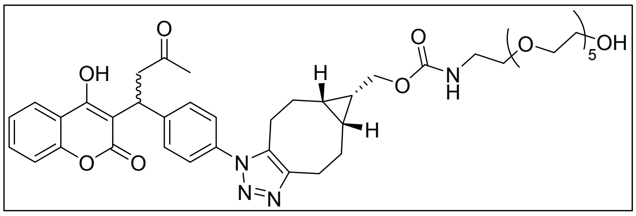

To a solution of **7** (1 eq., 80 mg, 0.175 mmol) in MeOH (3.5 mL) was added **2** (1.2 eq., 73.3 mg, 0.21 mmol). The reaction was stirred at room temperature for 6h. After concentration, the crude material was purified by silica gel flash chromatography (DCM to DCM/MeOH 85/15) to give clicked product **10** (98 % HPLC yield) as a yellow solid. **^{1}H NMR (400 MHz, $CDCl_3$):** δ 7.88 (d, *J* = 7.3 Hz, 1H), 7.49 (t, *J* = 7.2 Hz, 1H), 7.38 (d, *J* = 7.8 Hz, 2H), 7.28 – 7.20 (m, 4H), 5.43 (s, 1H), 4.26 (s, 1H), 4.12 (d, *J* = 7.6 Hz, 2H), 3.73 – 3.67 (m, 2H), 3.67 – 3.55 (m, 20H), 3.56 – 3.48 (m, 2H), 3.40 – 3.30 (m, 2H), 3.25 – 3.06 (m, 1H), 2.98 – 2.81 (m, 2H), 2.71 – 2.52 (m, 1H), 2.29 – 2.18 (m, 1H), 2.18 – 2.06 (m, 1H), 1.81 (s, 3H), 1.60 – 1.44 (m, 2H), 1.35 – 1.17 (m, 3H), 1.13 – 1.03 (m, 1H), 1.03 – 0.91 (m, 1H). **^{13}C NMR (125 MHz, $CDCl_3$)**: ^{13}C NMR (126 MHz, $CDCl_3$) δ 155.9, 152.0, 143.8, 133.7, 133.5, 130.7, 127.4, 127.3, 124.8, 122.8, 122.2, 115.4, 76.3, 76.0, 75.8, 71.5, 69.5 – 69.41, 69.1, 61.5, 60.5, 39.8, 34.3, 34.2, 30.9, 28.7 – 28.6, 28.4, 24.9, 24.86, 22.6, 22.5, 21.8, 21.8, 21.7, 21.6, 21.51, 19.1 – 18.9, 16.9, 13.1. **HRMS (ESI, m/z):** calcd for $C_{42}H_{55}N_4O_{12}$ $[M+H]^+$ 807.3811; found 807.3836.

NMR and HRMS analyses of WN_3 (2), BCN-peg$_6$-OH (9) and clicked product (10)

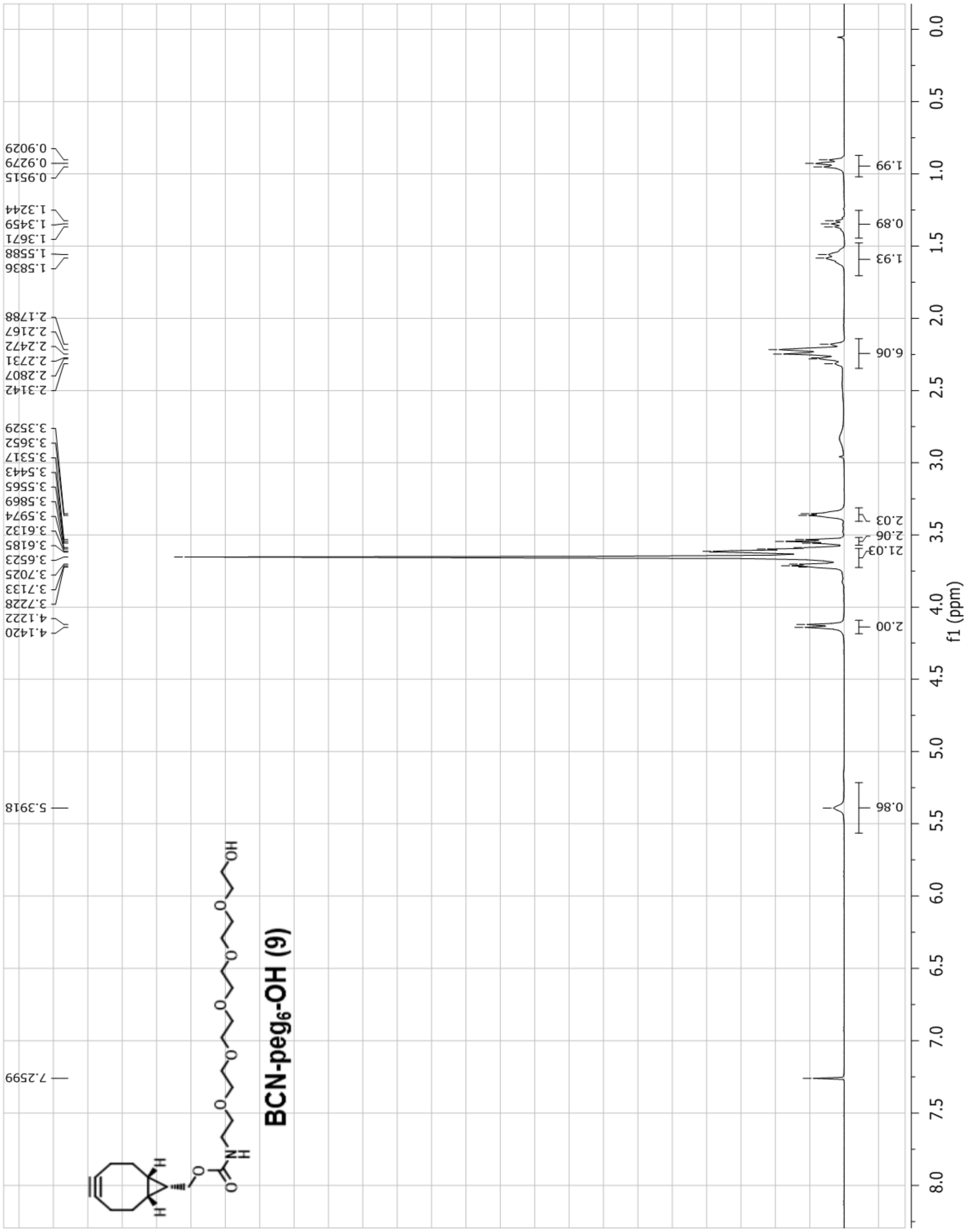

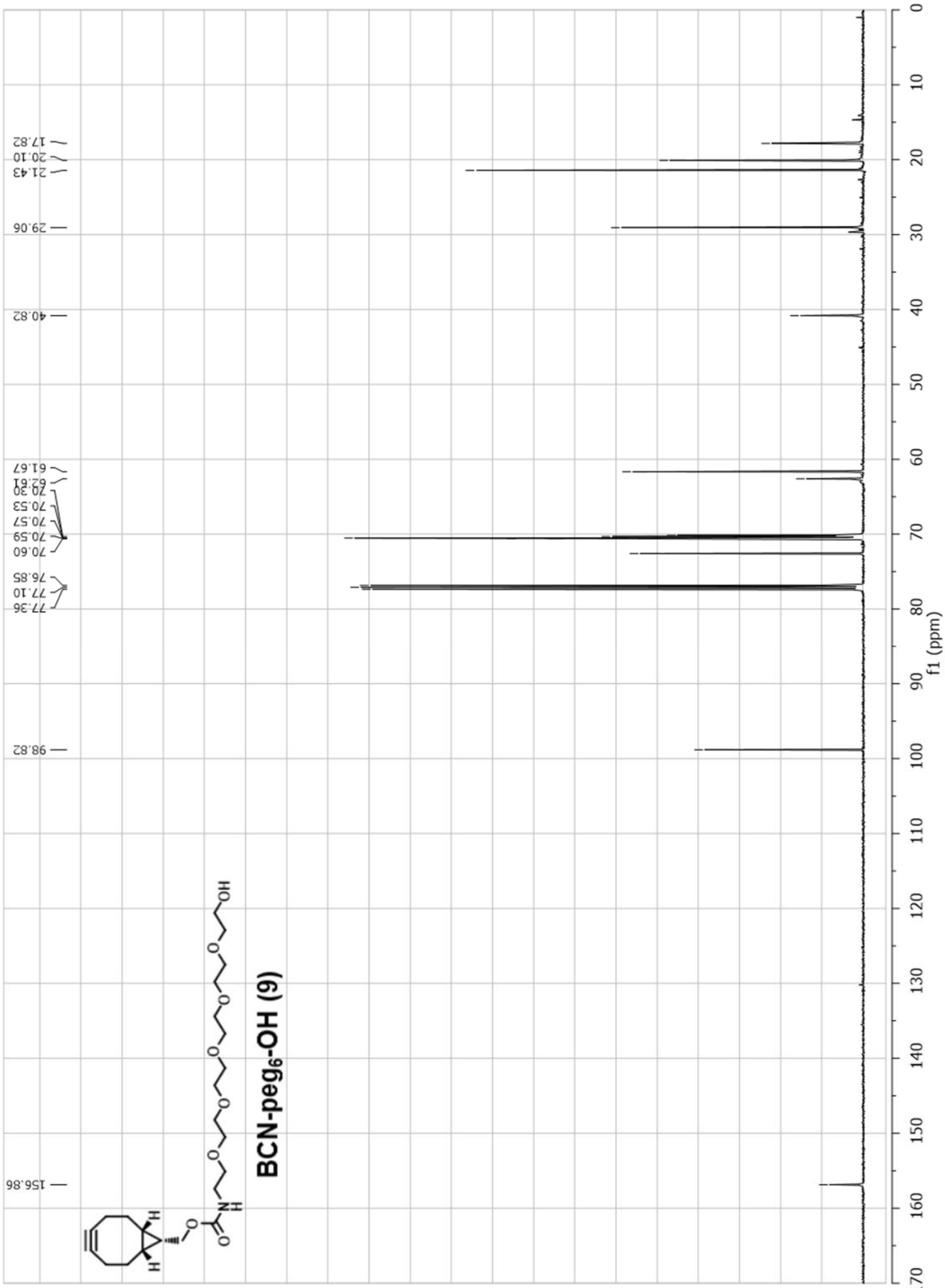

Supplementary Figure 1. ^{1}H and ^{13}C NMR spectra of BCN-peg$_{6}$-OH (9).

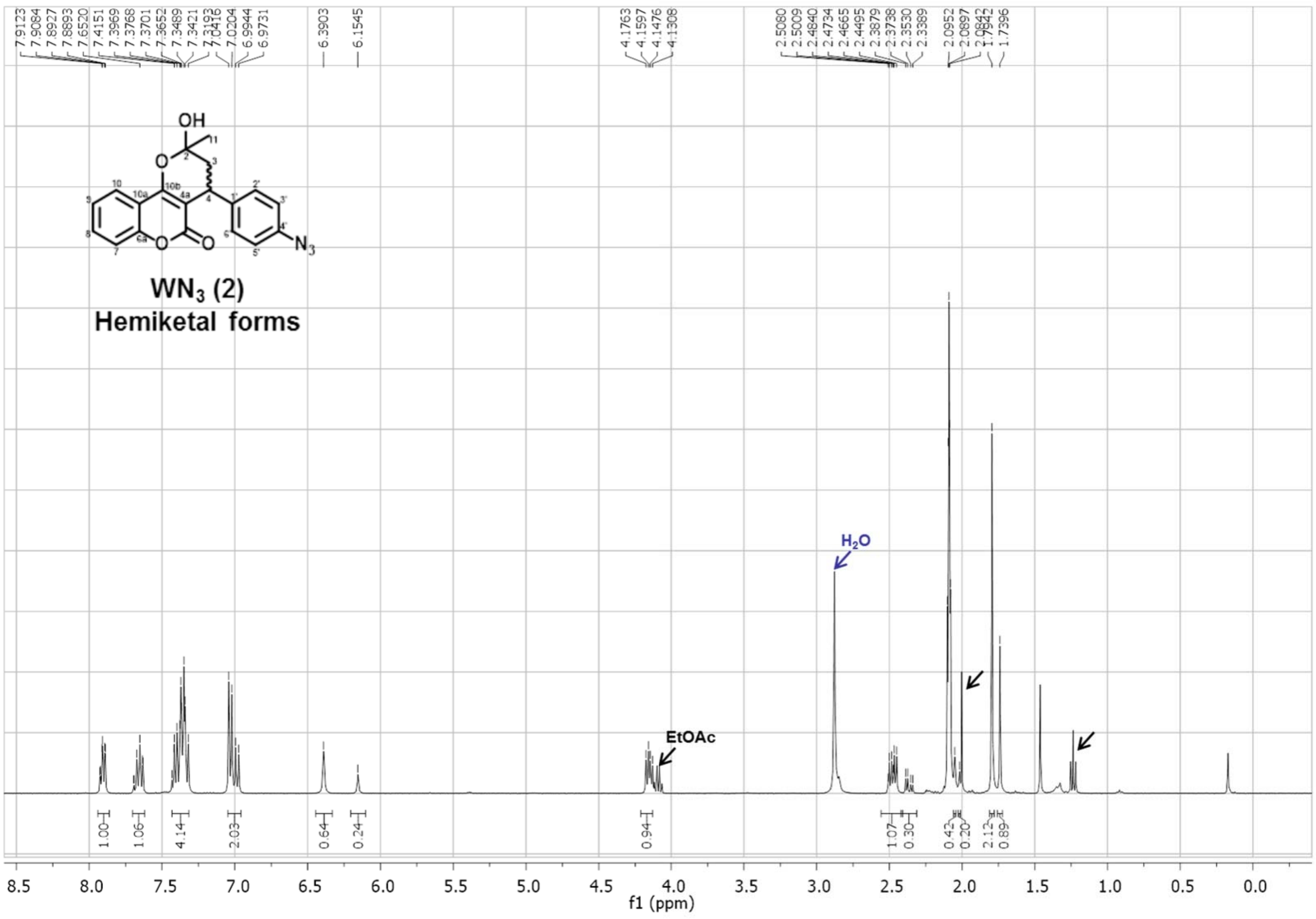

7.9123
7.9084
7.8927
7.8893
7.6520
7.4151
7.3969
7.3768
7.3701
7.3652
7.3489
7.3421
7.3193
7.0416
7.0204
6.9944
6.9731
6.3903
6.1545
4.1763
4.1597
4.1476
4.1308
2.5080
2.5009
2.4840
2.4734
2.4665
2.4495
2.3879
2.3738
2.3530
2.3389
2.0952
2.0897
2.0842
1.7942
1.7396
OH
WN3 (2)
Hemiketal forms
H2O
EtOAc
1.00
1.06
4.14
2.03
0.64
0.24
0.94
1.07
0.30
0.42
0.20
2.12
0.89
8.5
8.0
7.5
7.0
6.5
6.0
5.5
5.0
4.5
4.0
3.5
3.0
2.5
2.0
1.5
1.0
0.5
0.0
f1 (ppm)

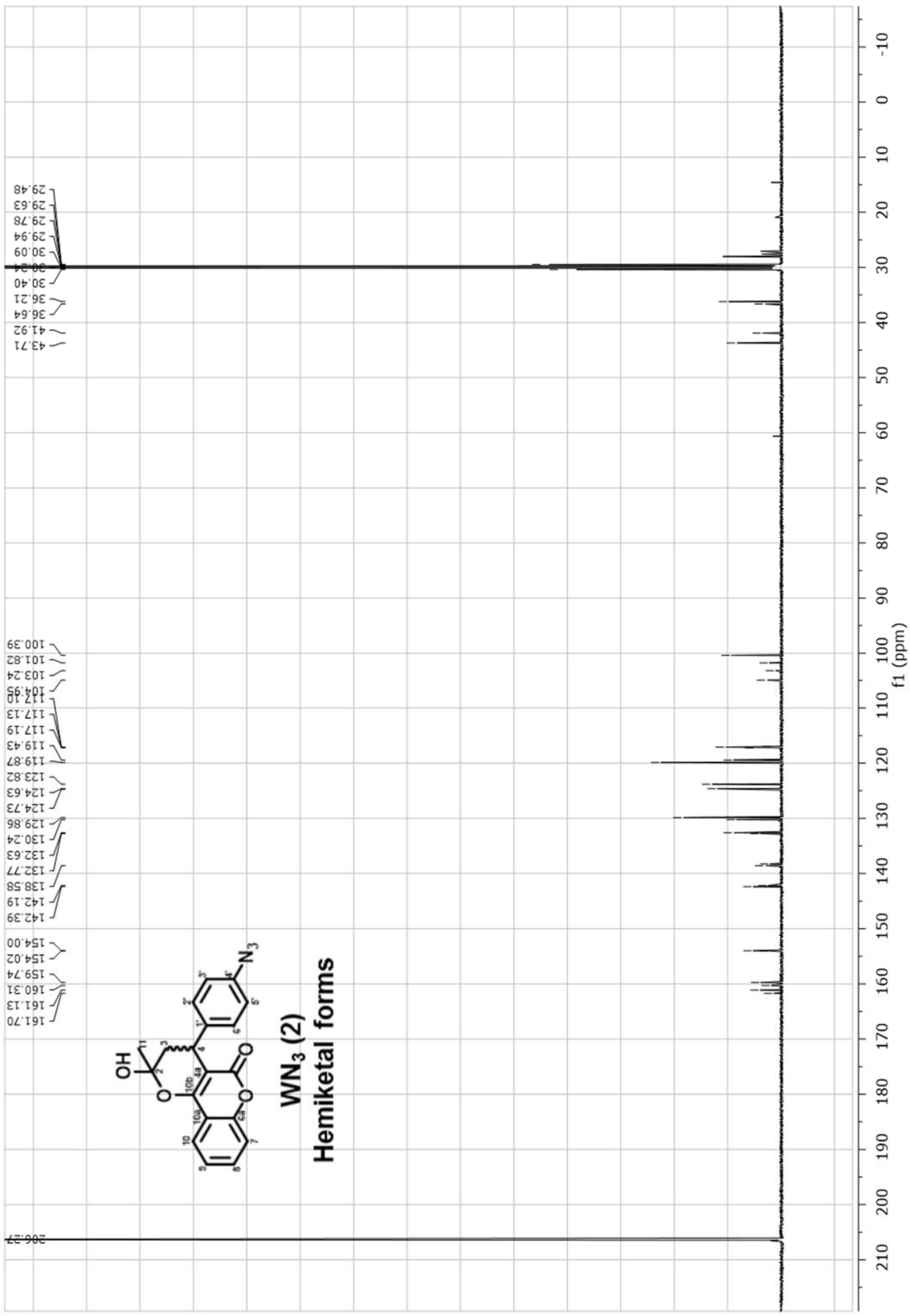

Supplementary Figure 2. ^{1}H and ^{13}C NMR spectra of WN_3 (2).

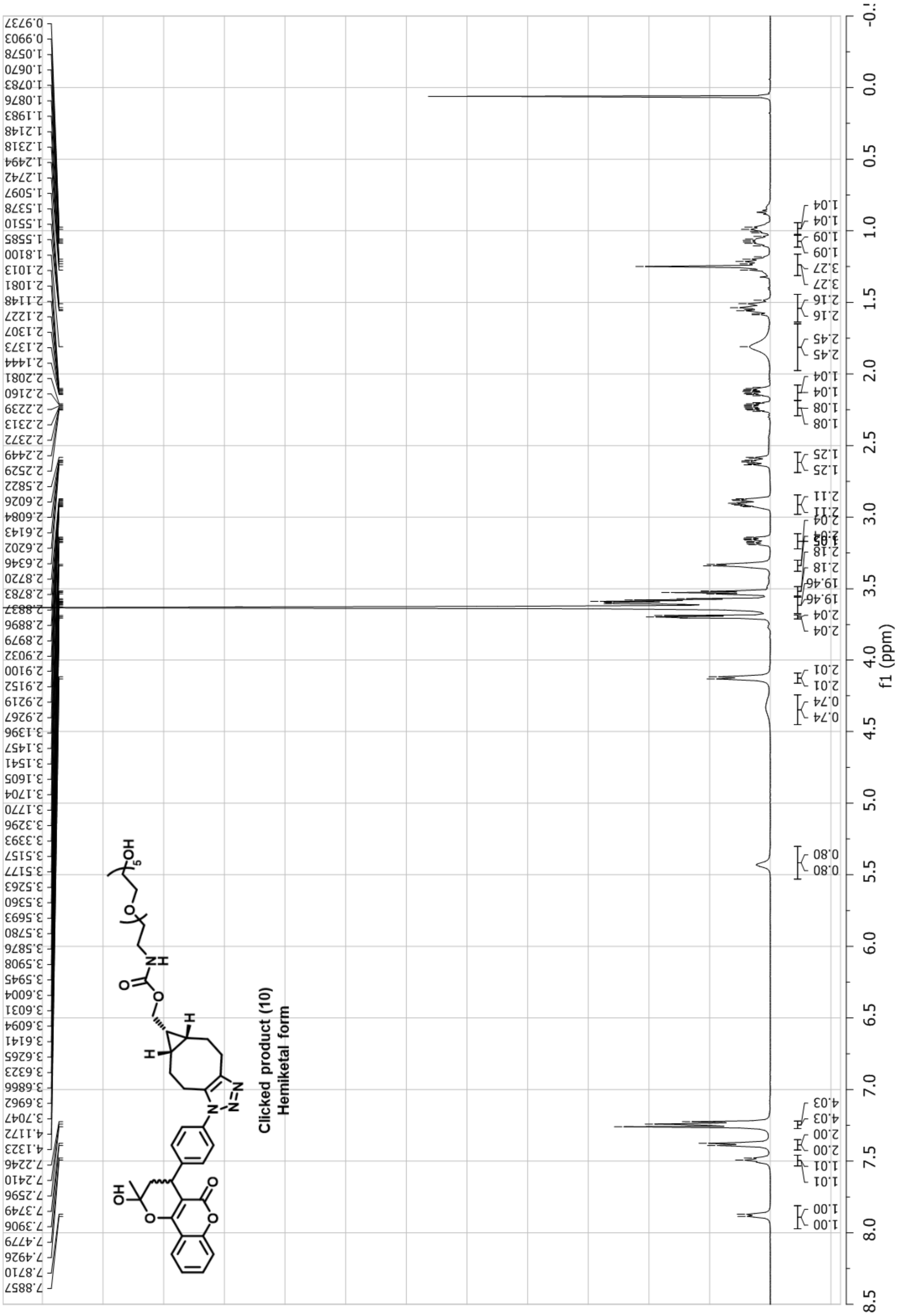
0.9737
0.9903
1.0578
1.0670
1.0783
1.0876
1.1983
1.2148
1.2318
1.2494
1.2742
1.5097
1.5378
1.5510
1.5585
1.8100
2.1013
2.1081
2.1148
2.1227
2.1307
2.1373
2.1444
2.2081
2.2160
2.2239
2.2313
2.2372
2.2449
2.2529
2.5822
2.6026
2.6084
2.6143
2.6202
2.6346
2.8720
2.8783
2.8837
2.8896
2.8979
2.9032
2.9100
2.9152
2.9219
2.9267
3.1396
3.1457
3.1541
3.1605
3.1704
3.1770
3.3296
3.3393
3.5157
3.5177
3.5263
3.5360
3.5693
3.5780
3.5876
3.5908
3.5945
3.6004
3.6031
3.6094
3.6141
3.6265
3.6323
3.6866
3.6962
3.7047
4.1172
4.1323
7.2246
7.2410
7.2596
7.3749
7.3906
7.4779
7.4926
7.8710
7.8857
Clicked product (10)
Hemiketal form
f1 (ppm)

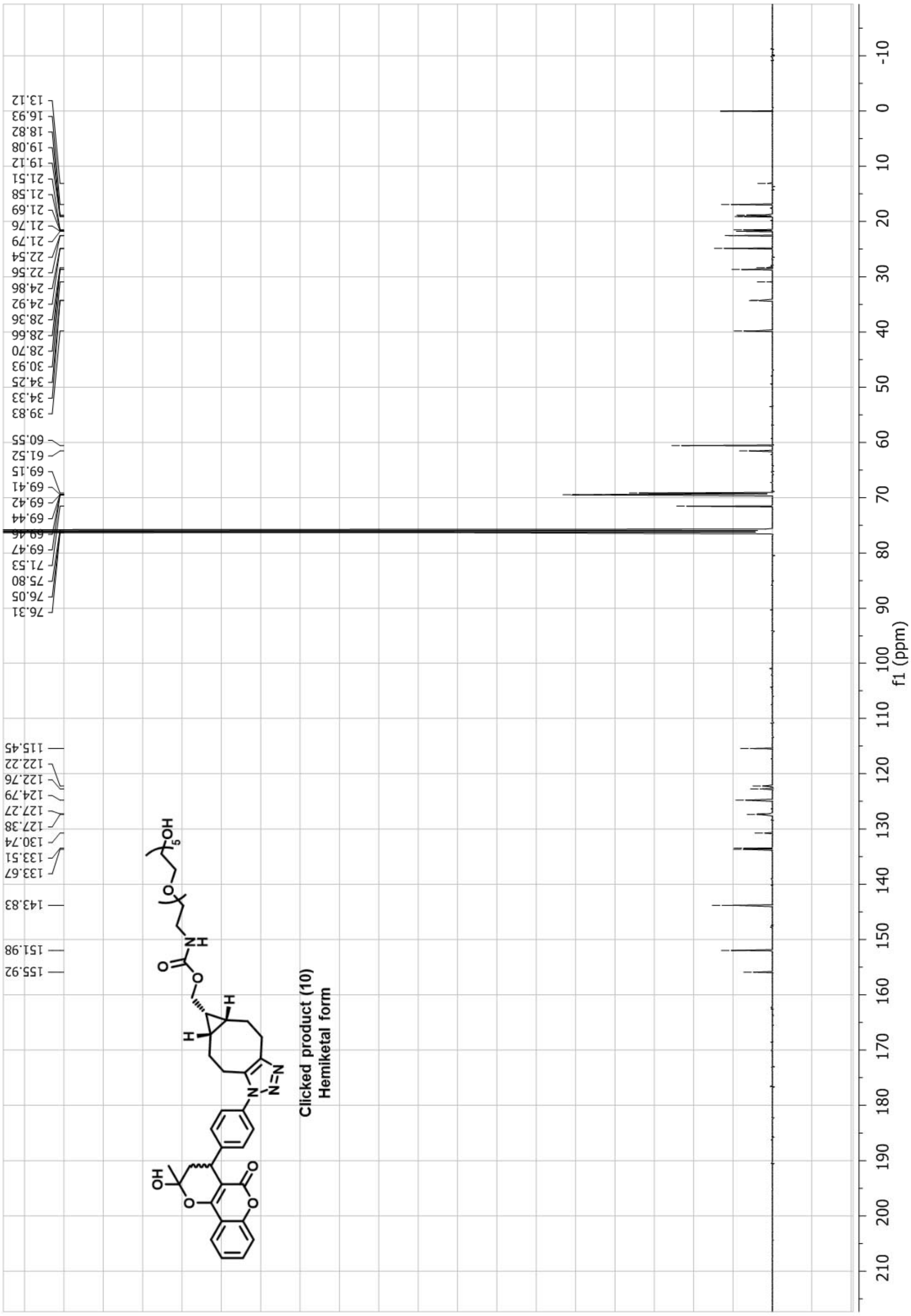

Supplementary Figure 3. ^{1}**H and** ^{13}**C NMR spectra of clicked product (10)**

BCN-peg$_6$-OH (9) HRMS

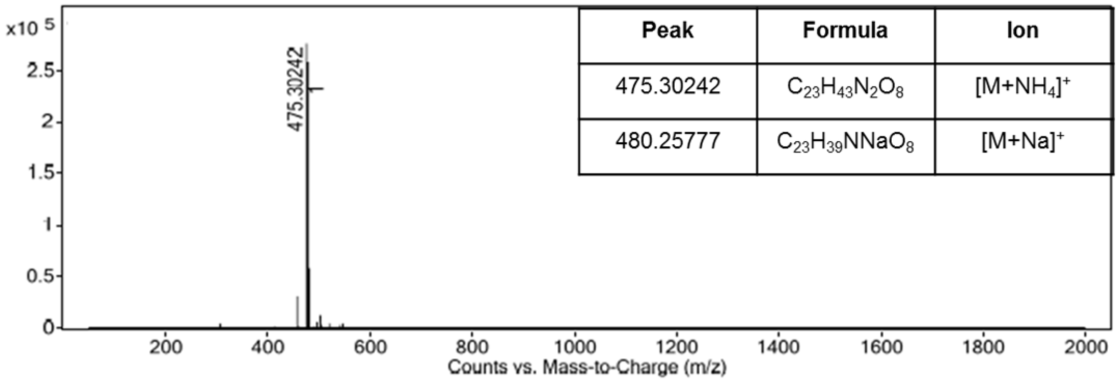

Supplementary Figure 4. BCN-peg$_6$-OH (9) HRMS spectra.

WN$_3$ (2) HRMS

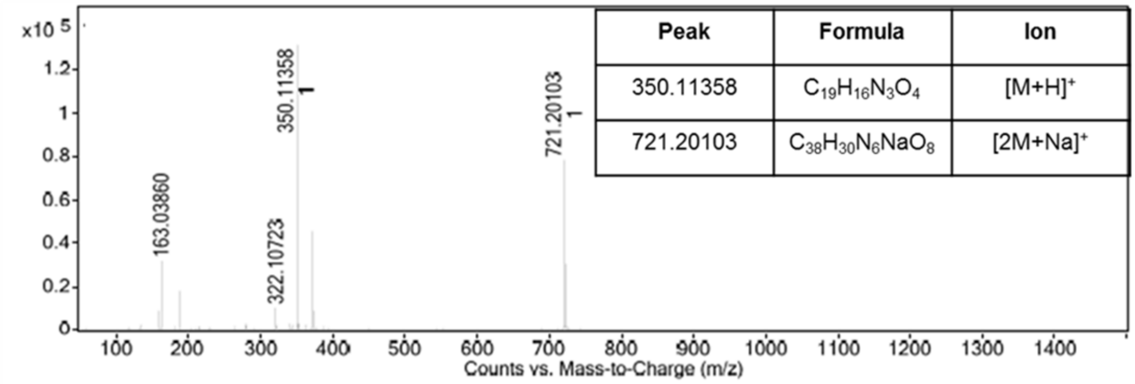

Supplementary Figure 5. WN$_3$ (2) HRMS spectra.

Clicked product (10) HRMS

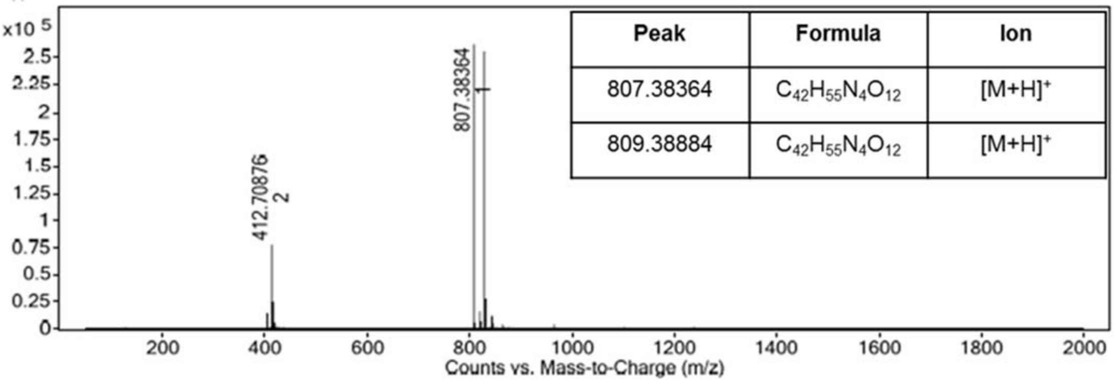

Supplementary Figure 6. Clicked product (10) HRMS spectra.

Kinetic studies in human plasma

Plasmatic samples extraction

The reactivity of WN_3 **2** and BCN-peg$_6$-OH **9** in human plasma was monitored by HPLC and normalized using Warfarin **1a** as internal standard. Collected plasmatic samples were analyzed after prior protein precipitation in cold acetonitrile. As WN_3 **2** and Warfarin **1a** (internal standard) are highly protein-bound, it was necessary to check that the extraction process proceeds efficiently and allows to extract warfarin derivatives even if they are bound to protein plasmatic.

Warfarin **1a**, WN_3 **2** and clicked product **10** were mixed with equimolar concentrations (100 µM) in human plasma and incubated at 37°C for 1h. To a 20 µL sample of plasma was added 80 µL of cold acetonitrile (d = 5, [**1a**] = [**2**] = [**10**] = 20 µM). The tube was centrifuged for 7 min at 4000 rpm and 50 µL of the supernatants were injected on HPLC (see Materials and Methods).
Standard samples were prepared using the same procedure starting from normal plasma samples. After precipitation, the resulting acetonitrile matrix (68.6 µL) was fortified with an equimolar solution of Warfarin **1a**, WN_3 **2** and BCN-peg$_6$-OH **9** (1.4 µL, 1 M) to obtain the theoretical concentration of extraction samples (20 µM). 50 µL of this solution were injected on HPLC.
These experiments were performed in triplicate and peak areas corresponding to the three compounds were measured and compared.

For the three compounds, the peak areas of extraction samples were not significantly different from those obtained for control experiments (Supplementary Figure 7) demonstrating that warfarin-derivatives can be completely extracted from plasma using this procedure. HPLC chromatograms obtained during extraction experiments are depicted in Supplementary Figure 8.

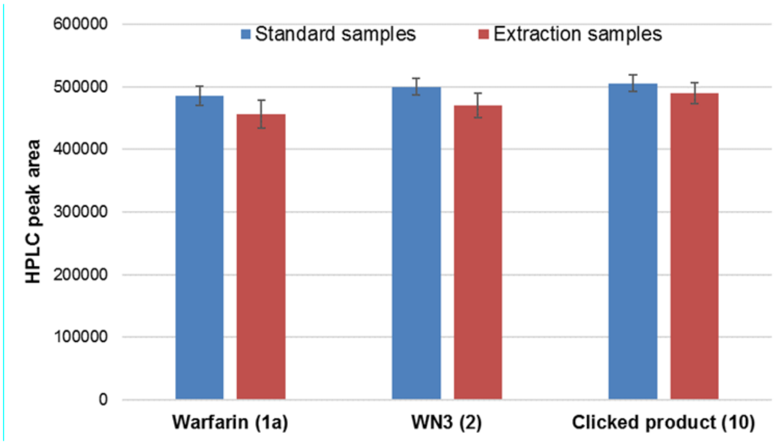

Supplementary Figure 7. Warfarin derivatives extraction from plamatic samples. Blue: HPLC peak areas obtained for standard samples ([**1a**] = [**2**] = [**10**] = 20 µM). Red: HPLC peak areas obtained for extraction of plasma samples contatining corresponding amounts of standard samples (Theoritical concentration: [**1a**] = [**2**] = [**10**] = 20 µM). These experiments were performed in triplicate and error bars indicate standard deviation.

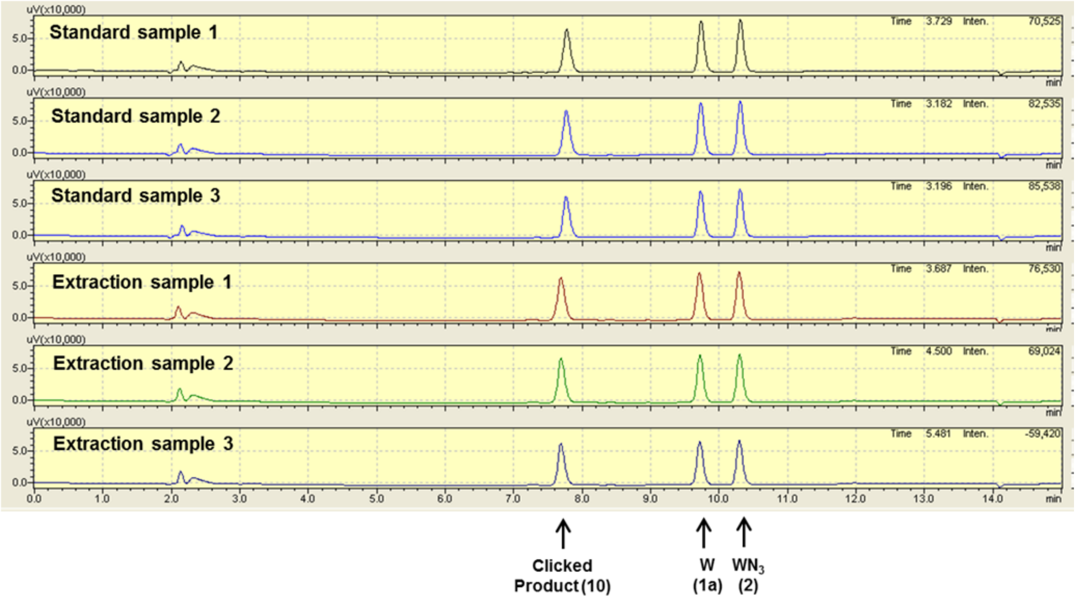

Supplementary Figure 8. HPLC chromatograms obtained during extraction experiments.

Clicked product (10) calibration curve

To evaluate accurately the click reaction in plasma, we monitored the formation of clicked product **10** and normalized its HPLC signal using Warfarin **1a** as internal standard. For this, solutions with increasing concentrations of clicked product **10** (0, 5, 10, 25, 50 and 75 and 100 μM) and Warfarin **1a** (100 μM) were prepared in DMSO. The resulting solutions were diluted in acetonitrile (d = 5) before HPLC injections to mimick the treatment of plasma samples during kinetic studies. The clicked product **10** concentrations were reported as the ratio of clicked product **10**/ Warfarin **1a** HPLC area peaks. From these data, a calibration curve of clicked product **10** concentrations was obtained (Supplementary Figure 9) and used during kinetic studies to determine the concentration of clicked product **10**. HPLC chromatograms recorded to establish the calibration curve of **10** are depicted in Supplementary Figure 10.

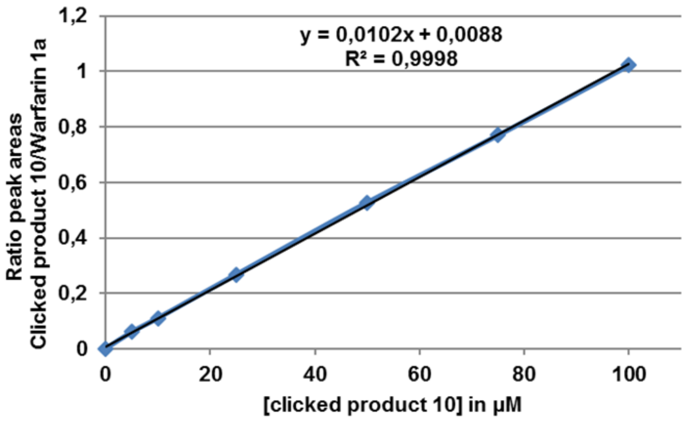

Supplementary Figure 9. Calibration curve of clicked product (10) concentrations using Warfarin (1a) as internal standard.

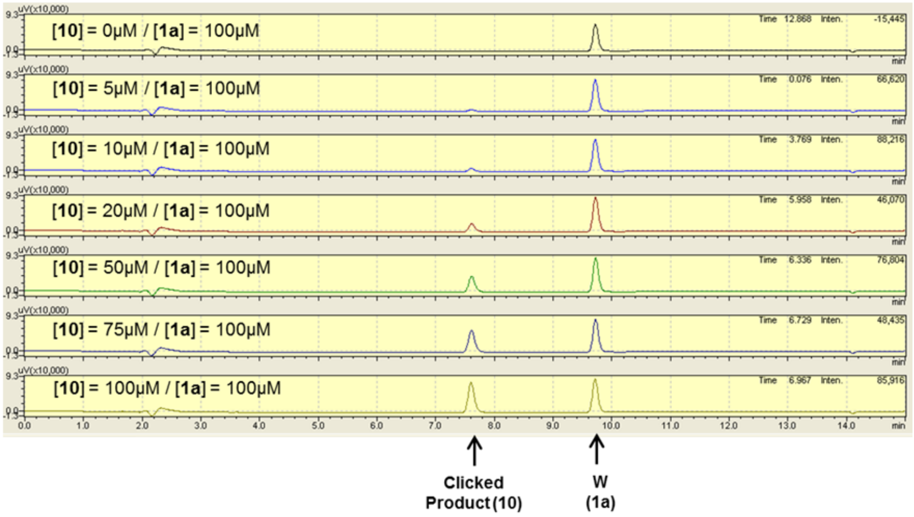

Supplementary Figure 10. HPLC chromatograms obtained during clicked product (10) calibration.

Second-order rate constant in plasma determination

Warfarin **1a**, WN_3 **2** and BCN-peg$_6$-OH **9** were mixed with equimolar concentrations (100 μM) in plasma and incubated at 37°C. Plasma samples (20 μL) were collected at regular time intervals (1, 15, 30, 45 and 60 minutes) and quenched with cold acetonitrile. After centrifugation (7 min at 4000 rpm), 50 μL of the supernatants were injected on HPLC (see Materials and Methods). This experiment was repeated in triplicate. HPLC spectra showed formation of the desired click adduct without formation of any byproducts (Supplementary Figure 11).

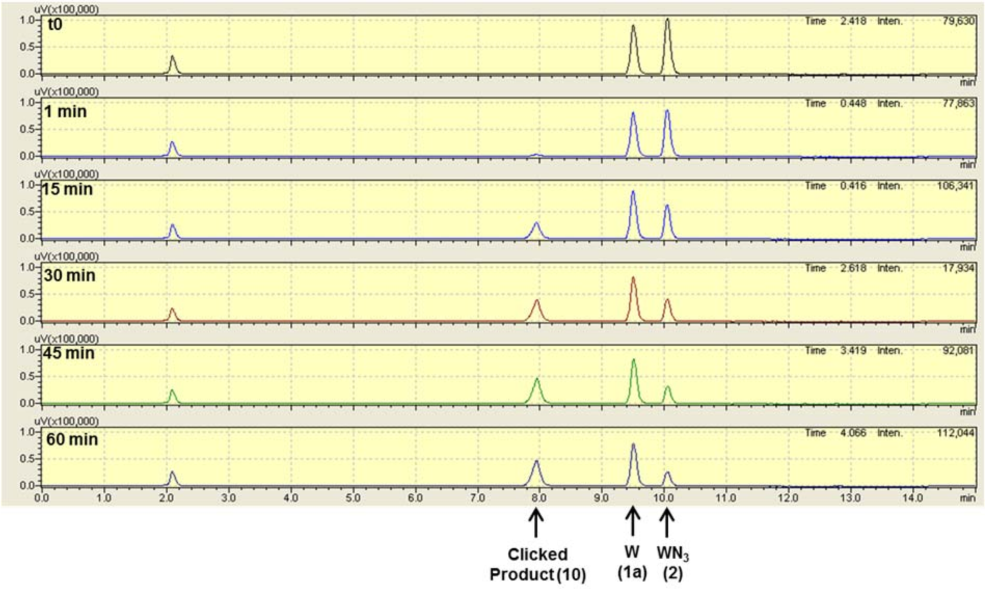

Supplementary Figure 11. HPLC monitoring of BCN-peg$_6$-OH/WN_3 click rection in human plasma.

To analyse click reaction, we monitored by HPLC the appearance of clicked product **10** and normalized its signal with that of Warfarin **1a**. The clicked product **10** concentration in plasma samples have been determined accurately by measuring the ratio of clicked product **10**/Warfarin **1a** HPLC peak area which has been then reported to the calibration curve (Supplementary Figure 9). Clicked product **10** conversion rate in plasma versus time was thus obtained (Supplementary Figure 12a). Second-order rate constants for the reaction were then determined by plotting the 1/[WN_3 2] versus time and analysis by linear regression (Supplementary Figure 12b). WN_3 **2** concentrations were calculated by subtracting the concentration of clicked product **10** formed from the initial concentration of WN_3 **2** (100 µM). A rate constant of 6.8 ± 1.8 $M^{-1}s^{-1}$ was found.

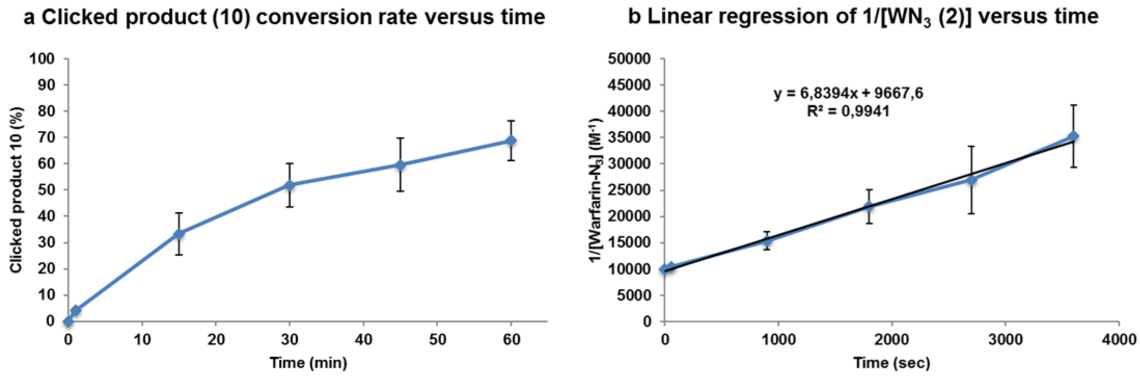

Supplementary Figure 12. Second-order rate constant determination in plasma. a, Conversion rate of clicked product **10** versus time. b, Linear regression of 1/[WN_3 **2**] versus time. These experiments were performed in triplicate and error bars indicate standard deviation.

WN_3 (2) and BCN-peg$_6$-OH (9) stability in plasma

The aging of WN_3 **2** and BCN-peg$_6$-OH **9** in human plasma was checked by recording kinetic experiments with sample of these reagents pre-incubated in plasma at 37°C.
A sample of WN_3 **2** kept at 37°C in plasma for 15 h led to a comparable kinetic of formation of clicked adduct when treated with a solution of BCN-peg$_6$-OH **9**. A rate constant of 5.1 ± 0.8 was found which is not significantly different than the result obtained with fresh plasmatic solution of WN_3 **2** (6.8 ± 1.8 $M^{-1}s^{-1}$). This indicates that i- WN_3 **2** did not decompose ii- WN_3 **2** is not sequestered by plasmatic protein and remains available for click reaction (Supplementary Figure 13).
Similarly, we then evaluated the effect of aging of BCN-peg$_6$-OH 9 in human plasma on its reactivity. We recorded kinetic experiments with a solution of BCN-peg$_6$-OH 9 pre-incubated for 15 h in human plasma at 37 °C. Reaction rate constant was found to be 6.8 ± 1.8 $M^{-1}s^{-1}$ with fresh solutions (Supplementary Figure 12b) and 2.4 ± 0.8 $M^{-1}s^{-1}$ (Supplementary Figure 14) after a 15 h preincubation. This decrease in reactivity indicates that BCN slowly looses its reactivity probably via deactivation through reaction with albumin SH group. However, considering the time of residence of BCN-peg$_6$-OH **9** in the mice organism (less than one hour, see Figure 7), we considered this stability to be more that sufficient to engage click and clear experiments.

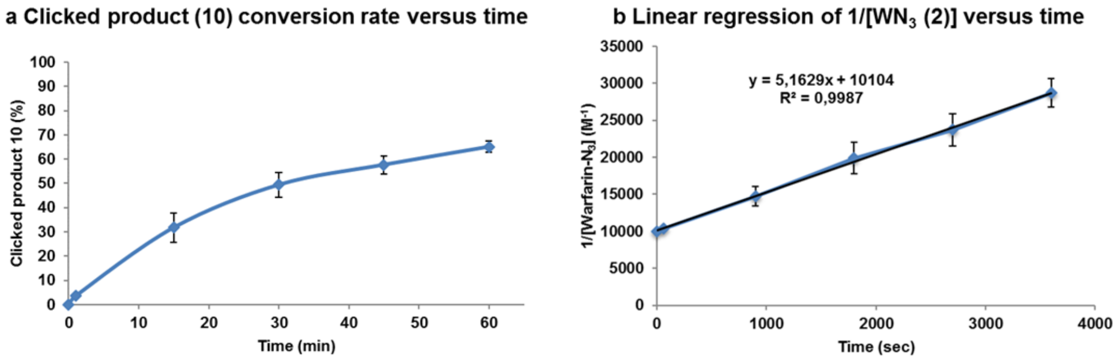

Supplementary Figure 13. Kinetic experiment after WN_3 (2) pre-incubation in plasma for 15h. a, Conversion rate of clicked product **10** versus time. b, Linear regression of 1/[WN_3 **2**] versus time. These experiments were performed in triplicate and error bars indicate standard deviation.

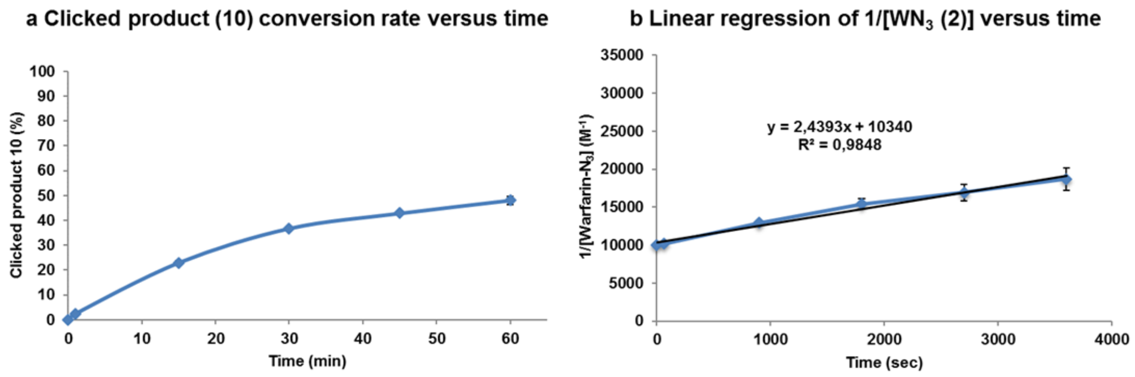

Supplementary Figure 14. Kinetic experiment after BCN-peg$_6$-OH (9) pre-incubation in plasma for 15h. a, Conversion rate of clicked product **10** versus time. b, Linear regression of 1/[WN_3 **2**] versus time. These experiments were performed in triplicate and error bars indicate standard deviation.

Anti-coagulant activity additionnal data

Procedure

Blood samples were centrifuged at room temperature for 2 minutes minimum 10 minutes after blood collection to recover plasma samples which were then analyzed by using Start4® system (Diagnostica Stago). Start4® is a benchtop hemostasis analyzer using mechanical clot detection. This viscosity-based detection system uses a magnetic bead which will be trapped after plasma sample coagulation. Prothrombine time measures were performed according to Diagnostica Stago guidelines for clinical uses. Briefly, 50 µL of each plasma sample were incubated at 37°C for 2 minutes in an appropriate test tube (Start4 cuvette, Ref. 38876 Diagnostica Stago) with a magnetic bead (Ref. 26441 Diagnostica Stago). Addition of calcium thromboplastine (100 µL, Neoplastine, Ref.00375 Diagnostica Stago) starts the timer, which would stop once the magnetic bead would be trap, related to the plasma sample coagulation.

Parent warfarin anticoagulant activity

The prothrombin time measurement was first validated after *per-os* administration (10 mg.kg^{-1}), twice with a 24-hour gap, n = 4-5) of native Warfarin **1a** and WN_3 **2** analogue (Supplementary Figure 15).

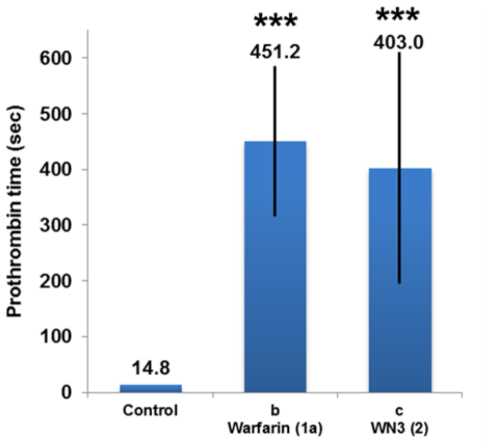

Supplementary Figure 15. In vivo biological activity of Warfarin (1a) and WN_3 (2). Prothrombin time was measured 24 hours after the second administration. **a**, no treated mice as control group, n = 6. **b**, Warfarin **1a** *per-os* administration (10 mg.kg^{-1}, twice with a 24-hour gap, n = 5). **c**, WN_3 **2** *per-os* administration (10 mg.kg^{-1}, twice with a 24-hour gap, n = 4). ***, $p<0.001$ as compared to control. Error bars indicate SEM values.

To demonstrate the absence of endogenous activity of BCN-peg$_6$-OH **9** on coagulation and thus further highlight the specificty of BCN-peg$_6$-OH **9** for an azide-modified target, we have tested that BCN-peg$_6$-OH **9** fails to reverse the anticoagulant effect of Warfarin (Supplementary Figure 16). Mice were treated with parent Warfarin compound (2 IP injections, 2.0 mg.kg^{-1}) followed by BCN-peg$_6$-OH **9** administration (SC injections, 52 mg.kg^{-1}, 20 eq) and effect on PT was assessed.

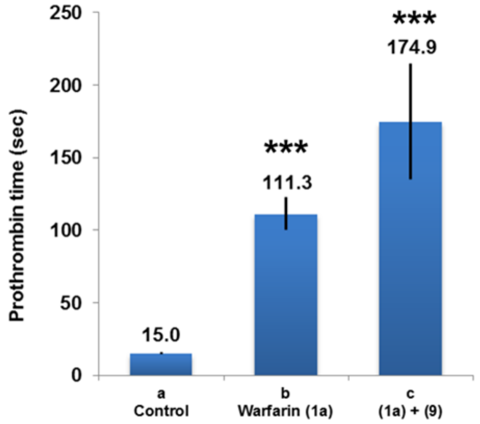

Supplementary Figure 16. Control experiment Warfarin (1a) vs Warfarin (1a) + BCN-peg$_6$-OH (9). a, vehicle IP injections to establish control group of mice (n = 4). **b,** Animal treated only with Warfarin **1a** (2.0 mg.kg^{-1}, 5.7 µmol.kg^{-1}, 1 eq) twice with a 24-hour gap by IP injection (n = 4). **c,** Warfarin **1a** IP injections (2.0 mg.kg^{-1}, 5.7 µmol.kg^{-1}, 1 eq) were followed by SC injections of BCN-peg6-OH **9** (52 mg.kg^{-1}, 114.4 µmol.kg^{-1}, 20 eq) 5 min post Warfarin **1a** delivery (n = 4). ***, p<0.001 as compared to control. Error bars indicate SEM values.

Inactivation of WN$_3$ (2) using vitamin K1 or BCN-peg$_6$-OH (9)

Controls were done to support that BCN-peg$_6$-OH **9** can inactive anticoagulant activity of WN$_3$ **2** and compared it to the activity of vitamin K1, the only treatment used in clinical conditions to reverse activity of Warfarin. First, vitamin K1 (SC injection, 20 mg.kg^{-1}) was validated to reverse WN$_3$ **2** anticoagulant activity. The PT values were very close from the control group (15.1 sec *vs* 15.0 sec for the control group, Supplementary Figure 17c). PT was also evaluated after SC injection of BCN-peg$_6$-OH 9 (SC injection, 52 mg.kg^{-1}) at different time-points post WN3 administration (5 min, 10 min and 30 min, Supplementary Figure 17d-f). The three experiments revealed a strong decrease of PT in accordance with the fact that WN$_3$ **2** is a circulating drug mostly localized in the plasma.

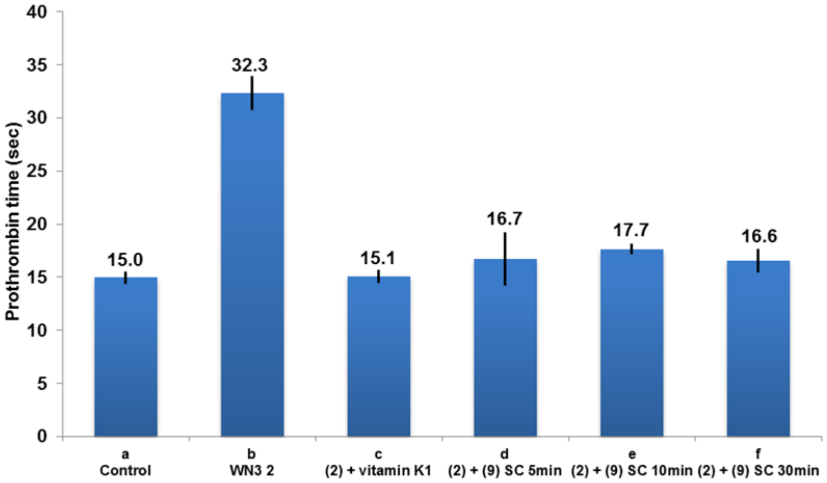

Supplementary Figure 17. Warfarin-N$_3$ (2) inactivation using vitamin K1 or BCN-peg$_6$-OH (9) post-WN$_3$ administration. a, vehicle IP injections to establish control group of mice (n = 4). **b,** Animal treated only with WN$_3$ **2** (2.0 mg.kg^{-1}, 5.7 µmol.kg^{-1}, 1 eq) twice with a 24-hour gap by IP injection (n = 4). **c,** WN$_3$

2 IP injections (2.0 mg.kg^{-1}, 5.7 µmol.kg^{-1}, 1 eq) were followed by SC injections of vitamin K (20 mg.kg^{-1}, 44.4 µmol.kg^{-1}, 8 eq) 5 min post WN_3 **2** delivery (n = 4). **d**, WN_3 **2** IP injections (2.0 mg.kg^{-1}, 5.7 µmol.kg^{-1}, 1 eq) were followed by SC injections of BCN-peg6-OH **9** (52 mg.kg^{-1}, 114.4 µmol.kg^{-1}, 20 eq) 5 min post WN_3 **2** delivery (n = 4). **e**, WN_3 **2** IP injections (2.0 mg.kg^{-1}, 5.7 µmol.kg^{-1}, 1 eq) were followed by SC injections of BCN-peg6-OH **9** (52 mg.kg^{-1}, 114.4 µmol.kg^{-1}, 20 eq) 10 min post WN_3 **2** delivery (n = 3). **f**, WN_3 **2** IP injections (2.0 mg.kg^{-1}, 5.7 µmol.kg^{-1}, 1 eq) were followed by SC injections of BCN-peg6-OH **9** (52 mg.kg^{-1}, 114.4 µmol.kg^{-1}, 20 eq) 30 min post WN_3 **2** delivery (n = 4). Error bars indicate SEM values.

Coagulation factors II, VII and X evaluation

The effect of warfarin derivatives and *in vivo* click reaction on the activity of coagulation factors II, VII and X was evaluated using Start4® system (Diagnostica Stago). The assay consists of the measurement of the clotting time, in the presence of calcium thromboblastin (Néoplastine, Stago) and excessive ammounts of all the clotting factors with exception of the tested factor (II, VII or X), which is thus entirely derived from the tested sample. Accordingly, an increased PT time directly reflects reduction of the tested clotting factor.

Procedure: Blood samples were centrifuged at room temperature for 2 minutes minimum 10 minutes after blood collection to recover plasma samples which were then diluted in STA-Owren Koller buffer (Ref: 00360 Diagnostica Stago). Briefly, 50 µL of the diluted plasma sample and 50 µL of the plasma deficient in one of the coagulation factor (STA-deficient II, VII or X) were incubated at 37°C for 60 sec in an appropriate test tube (Start4 cuvette, Ref. 38876 Diagnostica Stago) with a magnetic bead (Ref. 26441 Diagnostica Stago). Addition of calcium thromboplastine (100 µL, Neoplastine, Ref.00375 Diagnostica Stago) starts the timer, which would stop once the magnetic bead would be trap, related to the plasma sample coagulation.

To determine the level of each coagulation factor we have next established calibration curves by plotting PT values obtained for each factor with increasing dilutions (1/10, 1/20, 1/40 and 1/80) of normal human plasma (Supplementary Figure 18). Concentrations of factors were calculated for experimental samples as percent of human plasma using calibration curve equiations shown in corresponding panels.

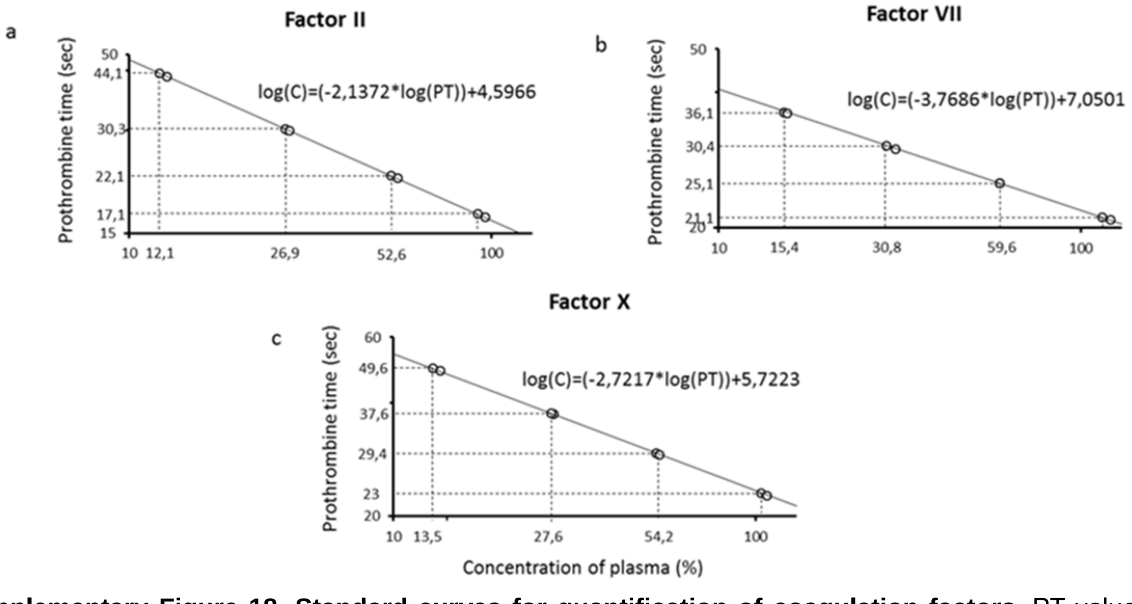

Supplementary Figure 18. Standard curves for quantification of coagulation factors. PT values of diluted normal human plasma were determined with kits dedicated for measures of coagulation factors II, VII and X and were shown in logarithmic scale respectively in panels a, b and c. The equations identified

for each calibration curve were shown in corresponding panels and were used to calculate concentration of coagulation factors for each treatment condition in Figures S14 and S15.

These experiments were first validated with Warfarin **1a** which significantly reduced relative concentration of each factor as increases significantly the clotting time indicating reduced activity of all the three coagulation factors compared as the control group (Supplementary Figure 19) and illustrated by increased clotting times (see mean PT values whithin corresponding barrs). BCN-peg$_6$-OH **9** was also injected in mice treated with Warfarin **1a** as negative control. No decrease of the clotting time was observed after BCN-peg$_6$-OH **9** administration as the click reaction can not occur.

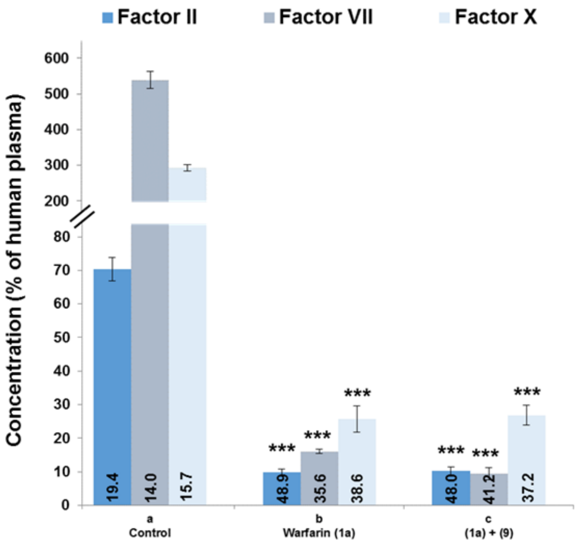

Supplementary Figure 19. Evaluation of coagulation factors II, VII and X after Warfarin (1a) treatment and BCN-peg$_6$-OH (9) injection. a, vehicle IP injections to establish control group of mice (n = 4). **b,** Animal treated only with Warfarin **1a** (2.0 mg.kg^{-1}, 5.7 µmol.kg^{-1}, 1 eq) twice with a 24-hour gap by IP injection (n = 4). **c,** Warfarin **1a** IP injections (2.0 mg.kg^{-1}, 5.7 µmol.kg^{-1}, 1 eq) were followed by SC injections of BCN-peg6-OH **9** (52 mg.kg^{-1}, 114.4 µmol.kg^{-1}, 20 eq) 5 min post Warfarin **1a** delivery (n = 4). Mean value of PT (in sec.) measures for each groupe was indicated whitin corresponding bar. ***, p<0.001 as compared to control. Error bars indicate SEM values.

The effect of WN$_3$ **2** and its inactivation was then studied. As Warfarin **1a**, WN$_3$ **2** significantly reduced concentration of all factors as illustrated by increased clotting times (see mean PT values whithin corresponding barrs). Injection of vitamin K1 or BCN-peg$_6$-OH **9**, post WN$_3$ **2** admistration, led to a strong decrease of the clotting time for the three factors indicating restration of activity of respective clotting factors (Supplementary Figure 20).

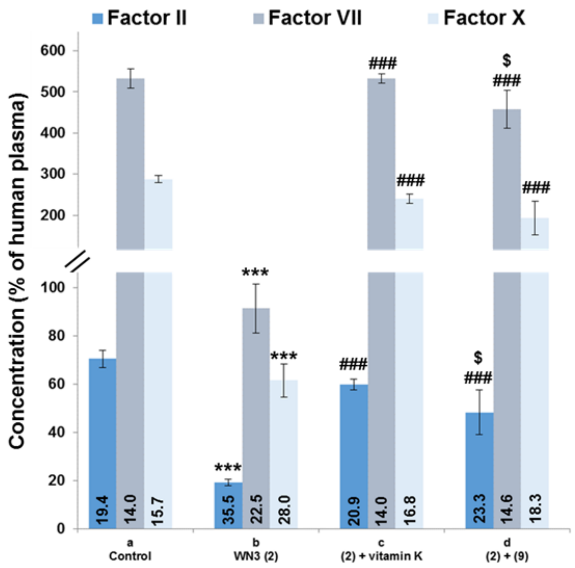

Supplementary Figure 20. Evaluation of coagulation factors II, VII and X after WN_3 (2) treatment and inactivation using vitamin K or BCN-peg$_6$-OH (9) post injection. a, vehicle IP injections to establish control group of mice (n = 4). **b,** Animal treated only with WN_3 **2** (2.0 $mg.kg^{-1}$, 5.7 $\mu mol.kg^{-1}$, 1 eq) twice with a 24-hour gap by IP injection (n = 4). **c,** WN_3 **2** IP injections (2.0 $mg.kg^{-1}$, 5.7 $\mu mol.kg^{-1}$, 1 eq) were followed by SC injections of vitamin K (20 $mg.kg^{-1}$, 44.4 $\mu mol.kg^{-1}$, 8 eq) 5 min post WN_3 **2** delivery (n = 4). **d**, WN_3 **2** IP injections (2.0 $mg.kg^{-1}$, 5.7 $\mu mol.kg^{-1}$, 1 eq) were followed by SC injections of BCN-peg6-OH **9** (52 $mg.kg^{-1}$, 114.4 $\mu mol.kg^{-1}$, 20 eq) 5 min post WN_3 **2** delivery (n = 4). Mean value of PT (in sec.) measures for each groupe was indicated whitin corresponding bar. ***, $p<0.001$ as compared with the corresponding factor measures in control group, ###, $p<0.001$ as compared with the corresponding factor measures in WN_3 **2** (**b**), $, $p<0.01$ as compared with the corresponding factor measures in **2** + vitamin K (**c**). Error bars indicate SEM values.

LC-MS/MS analyses of blood samples

Blood samples preparation: Plasma samples were first prepared by centrifugation at room temperature for 2 minutes, minimum 10 min after blood collection. To 20 µL of plasma samples were added 30 µL of acetonitrile and samples were centrifuged for 7 min at 4000 rpm in order to eliminate proteins. Supernatants (1 µL) were then analyzed by LC-MS/MS.

Calibration: The external standard technique was used for the quantification. Four plasma samples from mouse (V = 200 µL) were prepared with different appropriate concentrations of each compound from standard DMSO stock solutions, to establish the external calibration curves in plasma sample plotted using peak areas and concentrations. Before injection, acetonitrile (800 µL) was added and samples were centriguged for 7 minutes at 4000 rpm. Supernatants (1 µL) were then analyzed by LC-MS/MS (see 1. Material and methods: Instrumentation and methods associated).
.

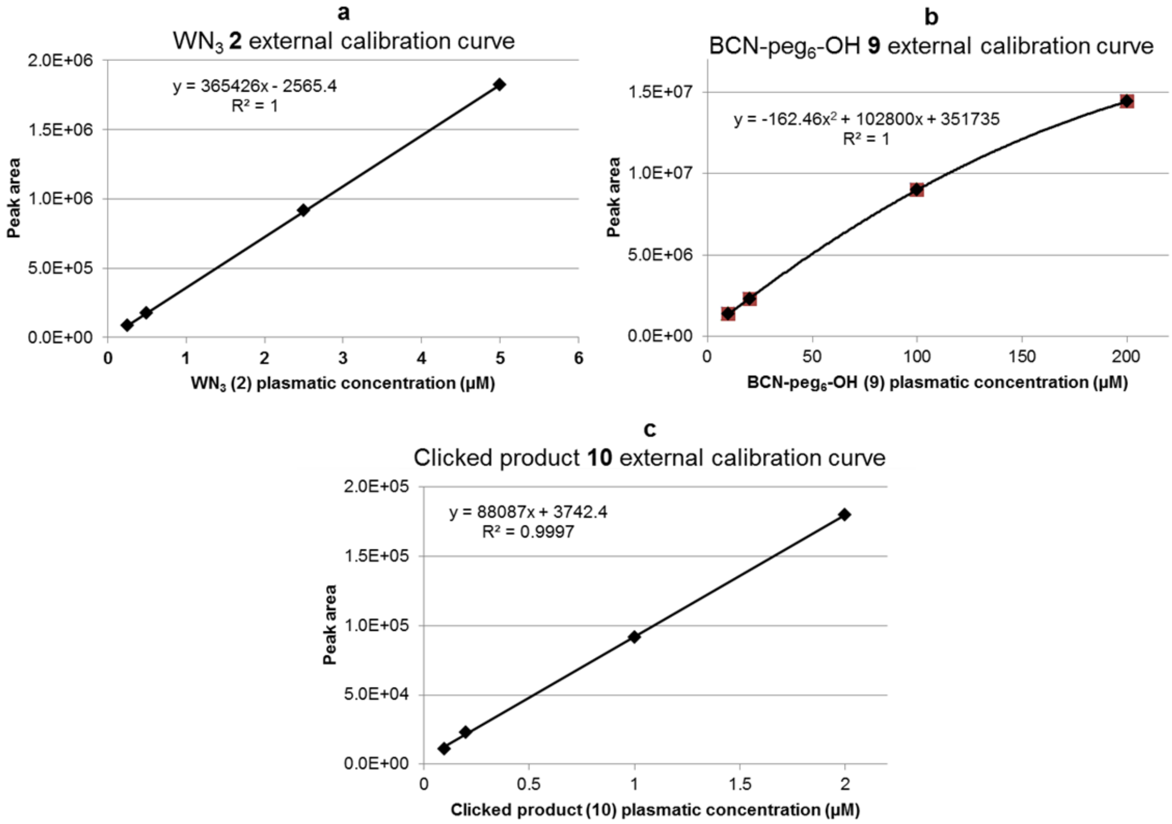

Supplementary Figure 21. External calibration curve in plasma of WN_3 (2), BCN-peg$_6$-OH (9) and clicked product (10). a, WN_3 **2** external calibration curve. **b**, BCN-peg$_6$-OH **9** external calibration curve. **c**, clicked product **10** external calibration curve.

LC-MS/MS analyses of urine samples

Urine sample preparation: 50 µL of an urine sample were diluted with water (200 µL) and the mixture was purified on a strata c18-E (55µm, 70A) SPE cartridge (8B-S001-DAK, Phenomenex) beforehand equilibrated with MeOH (1 mL) and water (1 mL). The cartridge is then washed with water and eluted with methanol (800 µL). The eluate was then dried under vacuum for 2 h and dissolved in H_2O/ACN (1/1, 500 µL). 0.5 µL of the solution was analyzed on LC-MS/MS.

Calibration: The external standard technique was used for the quantification. Four urine mice samples (V = 50 µL) were prepared with different appropriate concentrations of each compound from standard DMSO stock solutions, to establish the external calibration curves in urine sample plotted using peak areas and concentrations. The calibration samples were purified according to the same procedure as described above (SPE cartridge).

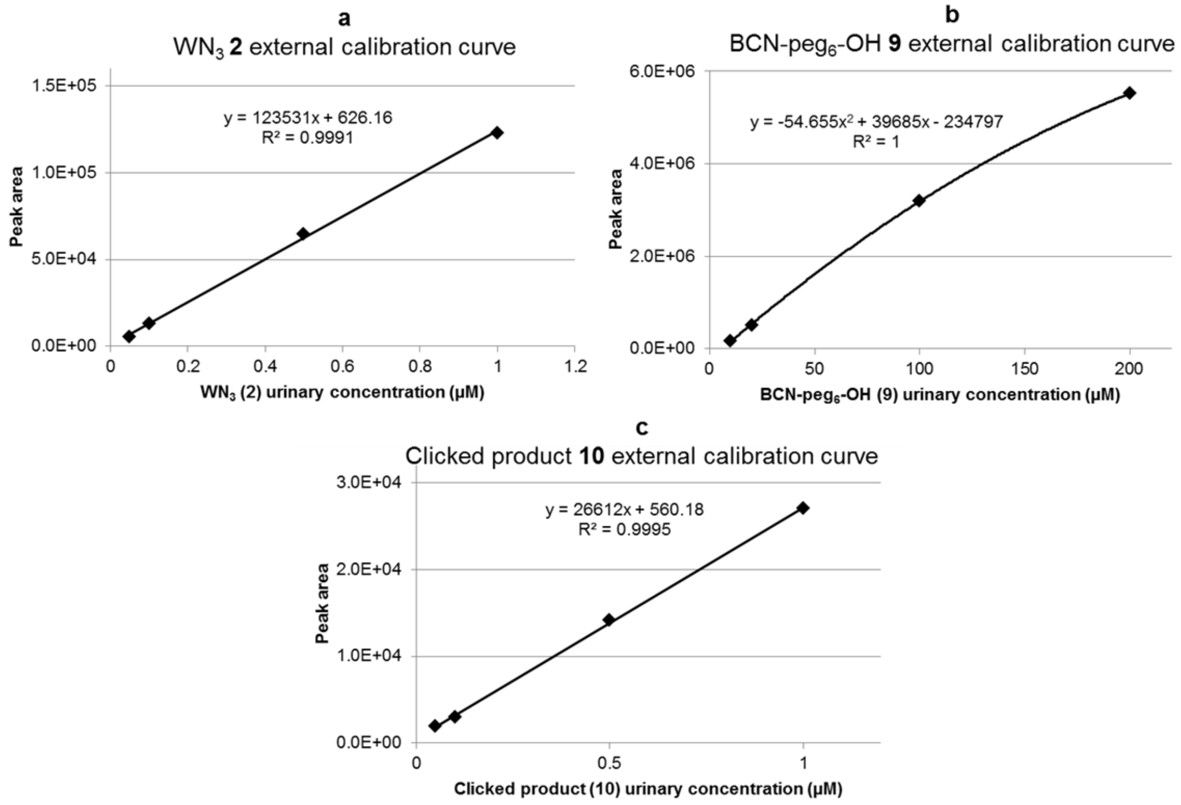

Supplementary Figure 22. External calibration curve in urine of WN_3 (2), BCN-peg$_6$-OH (9) and clicked product (10). a, WN_3 **2** external calibration curve. **b**, BCN-peg$_6$-OH **9** external calibration curve. **c**, clicked product **10** external calibration curve.

LC-MS/MS analyses of liver samples

Liver samples preparation: Liver samples were comminuted in a mixture of saline solution (400 µL) and acetonitrile (800 µL). The samples were vortexed, sonicated for 3 min and centrifuged (15 000 g, 5 min, 4°C). 0.5 µL of the supernatants were then analyzed by LC-MS/MS.

Calibration: Three liver samples were prepared with different appropriate concentrations of each compound from standard DMSO stock solutions, to establish the external calibration curves. The calibration samples were treated by using the same procedure as described above.

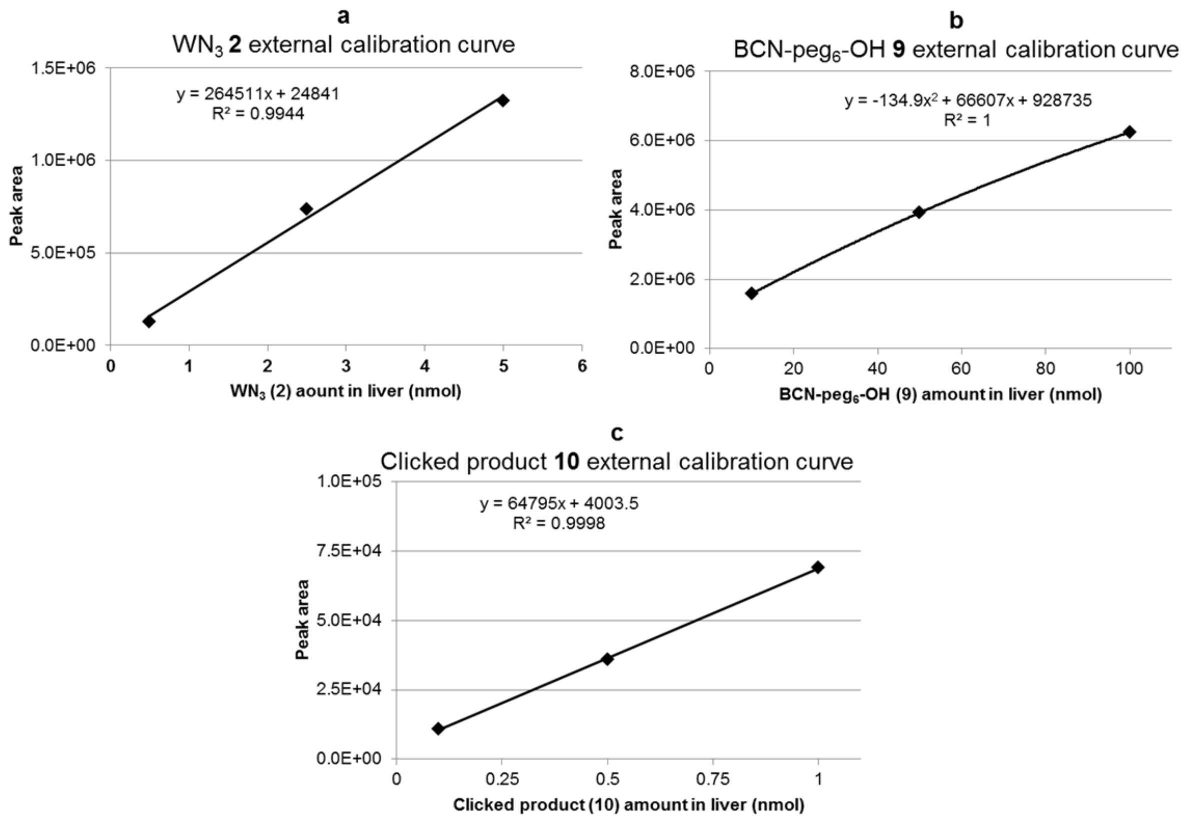

Supplementary Figure 23. External calibration curve in liver of WN_3 (2), BCN-peg$_6$-OH (9) and clicked product (10). a, WN_3 **2** external calibration curve. **b**, BCN-peg$_6$-OH **9** external calibration curve. **c**, clicked product **10** external calibration curve.

Plasmatic proteins binding of Warfarin derivatives

Plasmatic protein binding of Warfarin and WN_3 derivatives was assessed using the RED (Rapid Equilibrium Dialysis) device (90006, ThermoFisher).

Sample preparation (n = 2): A solution of 1 µM of the studied compound was prepared in a mixture of plasma and PBS 1x (1/1). 200 µL of this solution was introduced in the sample chamber and 350 µL of PBS 1x were introduced in the buffer chamber. The unit was covered with sealing tape and incubated at 37°C on an orbital shaker at 250 rpm for 4 h. Seal was removed and 50 µL samples of both the buffer and the plasma chambers were collected and placed in separate microcentrifuge tubes. An equal volume of plasma/PBS (1/1) and PBS were respectively added to the buffer and the sample chamber. ACN (200 µL) was added and samples were centriguged for 7 minutes at 15000 g and 4°C. Supernatants (2 µL) were then analyzed by LC-MS/MS.

The percentage of the test compound bound was calculated as follows:

% $Free_{50}$ = (Peak area of buffer chamber / Peak area of plasma chamber) × 100%

% $Free_{100}$ = % $Free_{50}$ / (2 - % $Free_{50}$)

% Bound = 100% - % Free

Both compounds were largely protein-bound: 94 ± 1% of Warfarin **1a** and 98 ± 1% of WN_3 **2** were recovered in the plasma chamber.

LC-MS/MS analyses recorded during plasmatic protein binding evaluation for Warfarin **1a** and WN_3 **2** are respectively reported in Supplementary Figure 24 and Supplementary Figure 25.

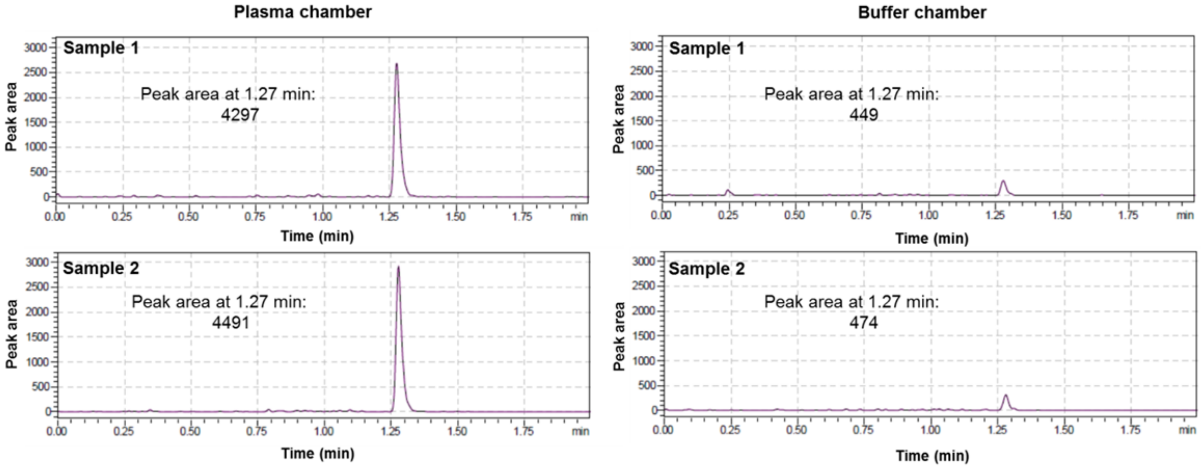

Supplementary Figure 24. LC-MS/MS analyses for evaluation of Warfarin (1a) plasmatic protein binding.

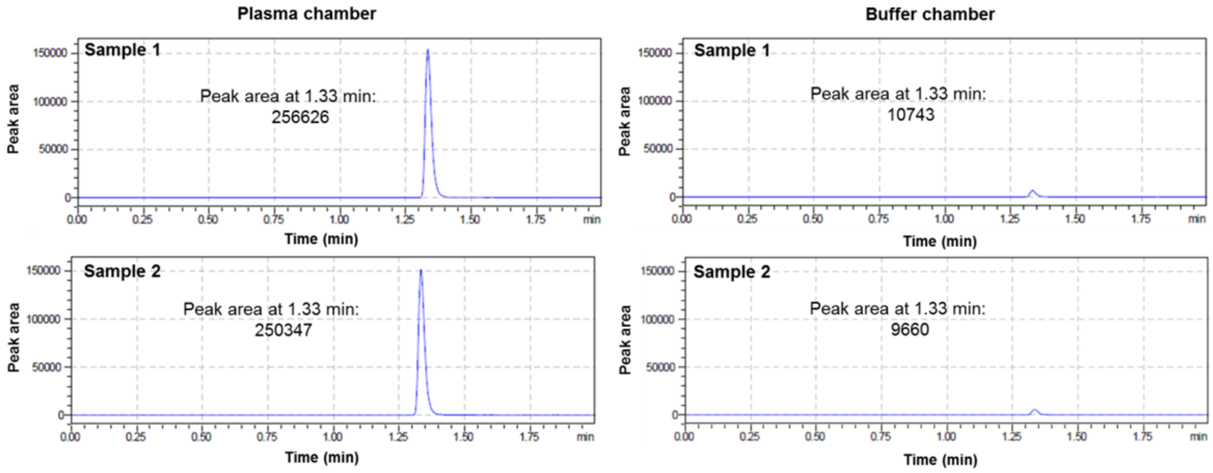

Supplementary Figure 25. LC-MS/MS analyses for evaluation of WN_3 (2) plasmatic protein binding

Supplementary References

[1] Zumbansen, K., Döhring, A. & List, B. Morpholinium trifluoroacetate-catalyzed aldol condensation of acetone with both aromatic and aliphatic aldehydes. *Adv. Synth. Catal.* **352**, 1135 (2010).

[2] Halland, N., Hansen, T. & Jorgensen, K. A. Organocatalytic asymmetric Michael reaction of cyclic 1,3-dicarbonyl compounds and α,β-unsaturated ketones - a highly atom-economic catalytic one-step formation of optically active warfarin anticoagulant. *Angew. Chem. Int. Ed.* **42**, 4955 (2003).

[3] Herner, A., Nikić, I., Kállay, M., Lemkeb E. A. & Kele, P. A new family of bioorthogonally applicable fluorogenic labels. *Org. Biomol. Chem.* **11**, 3297 (2013).

[4] Zumbansen, K., Döhring, A. & List, B. Morpholinium trifluoroacetate-catalyzed aldol condensation of acetone with both aromatic and aliphatic aldehydes. *Adv. Synth. Catal.* **352**, 1135 (2010).

[5] Pisklaka, M., Maciejewsk, D., Heroldc, F., Wawera, I. Solid state structure of coumarin anticoagulants: warfarin and sintrom. ^{13}C CPMAS NMR and GIAO DFT calculations. *J. Mol. Struct.* **649**, 169 (2003).

[6] Dommerholt, J., Schmidt, S., Temming, R., Hendriks, L. J. A., Rutjes, F. P. J. T., van Hest, J. C. M., Lefeber, D. J., Friedl, P. & van Delft, F. L. Readily accessible bicyclononynes for bioorthogonal labeling and three-dimensional imaging of living cells. *Angew. Chem. Int. Ed.* **49**, 9422 (2010).

[7] Bouzide, A. & Sauve G. Silver(I) oxide mediated highly selective monotosylation of symmetrical diols. Application to the synthesis of polysubstituted cyclic ethers. *Org Lett.* **4**, 2329 (2002).

[8] Müller, M. K., & Brunsveld, L. A supramolecular polymer as a self-assembling polyvalent scaffold. *Angew. Chem. Int. Ed.* **48**, 2921 (2009).

[9] Müller, M. K., Petkaub, K. & Brunsveld, L. Protein assembly along a supramolecular wire. *Chem. Commun.* **47**, 310 (2011).
